# Evaluating the implementation of the Primary Health Integrated Care Project for Chronic Conditions: a cohort study from Kenya

**DOI:** 10.1136/bmjph-2023-000146

**Published:** 2024-03-25

**Authors:** Richard Mugo, Triantafyllos Pliakas, Jemima Kamano, Leah Anku Sanga, Ellen Nolte, Antonio Gasparrini, Edwine Barasa, Anthony Etyang, Pablo Perel

**Affiliations:** 1 AMPATH Kenya, Eldoret, Kenya; 2 Department of Public Health, Environments and Society, London School of Hygiene & Tropical Medicine, London, UK; 3 GSK Vaccines, Wavre, Belgium; 4 School of Medicine, Moi University College of Health Sciences, Eldoret, Kenya; 5 London School of Hygiene & Tropical Medicine, London, UK; 6 Department of Health Services Research and Policy, London School of Hygiene & Tropical Medicine, London, UK; 7 Health Economics Research Unit, KEMRI-Wellcome Trust Research Programme Nairobi, Nairobi, Kenya; 8 KEMRI-Wellcome Trust Research Programme Nairobi, Nairobi, Kenya

**Keywords:** public health, epidemiology, public health practice

## Abstract

**Introduction:**

In Kenya, non-communicable diseases (NCDs) are estimated to account for almost one-third of all deaths and this is likely to rise by over 50% in the next 10 years. The Primary Health Integrated Care for Chronic Conditions (PIC4C) project aims to strengthen primary care by integrating comprehensive NCD care into existing HIV primary care platform. This paper evaluates the association of PIC4C implementation on clinical outcomes.

**Methods:**

Outcomes included proportion of new patients, systolic blood pressure (SBP), fasting plasma glucose (FPG), diastolic blood pressure, hypertension control, random plasma glucose, diabetes control, viral load and HIV viral suppression. We used interrupted time series and binomial regression with random effects for facility-level data and generalised mixed-effects regression for visit-level data to examine the association between PIC4C and outcomes between January 2017 and December 2021. We conducted sensitivity analysis with restrictions on sites and the number of visits.

**Results:**

Data from 66 641 visits of 13 046 patients with hypertension, 24 005 visits of 7267 patients with diabetes and 84 855 visits of 21 186 people with HIV were analysed. We found evidence of association between PIC4C and increase in proportion of new patients per month with hypertension (adjusted OR (aOR) 1.57, 95% CI 1.39 to 1.78) and diabetes (aOR 1.31, 95% CI 1.19 to 1.45), small increase in SBP (adjusted beta (aB) 1.7, 95% CI 0.8 to 2.7) and FPG (aB 0.6, 95% CI 0.0 to 1.1). There was no strong evidence of association between PIC4C and viral suppression (aOR 1.20, 95% CI 0.98 to 1.47). In sensitivity analysis, there was no strong evidence of association between PIC4C and SBP (aB 1.74, 95% CI −0.70 to 4.17) or FPG (aB 0.52, 95% CI −0.64 to 1.67)

**Conclusions:**

PIC4C implementation was associated with increase in proportion of new patients attending clinics and a slight increase in SBP and FPG. The immediate post-PIC4C implementation period coincided with the COVID-19 pandemic, which is likely to explain some of our findings.

**WHAT IS ALREADY KNOWN ON THIS TOPIC**

- Available evidence suggests that integrating non-communicable disease (NCD) and HIV care is feasible and can be effective in improving identification and management of people with hypertension and or diabetes.

**WHAT THIS STUDY ADDS**

- This is the largest study in SSA using electronic health records to examine the association of healthcare model that integrated NCD services into an existing primary care HIV platform, measuring effectiveness of integrating NCD and HIV services on hypertension, diabetes and HIV control which has not been done by other studies.

**HOW THIS STUDY MIGHT AFFECT RESEARCH, PRACTICE OR POLICY**

- The study provides some evidence of improvement in new patient recruitment during Primary Health Integrated Care for Chronic Conditions (PIC4C) implementation; however, it was associated with a small deterioration in hypertension and diabetes control. We did not find evidence that implementation of PIC4C had a negative effect on the HIV population attending these clinics. As part of a larger research programme a separate qualitative paper, with patients, healthcare providers and decision-makers will report potential explanations for our findings.

## Introduction

 Non-communicable diseases (NCDs), such as hypertension and diabetes, account for an estimated 41 million deaths globally each year, the majority (~80%) of which occur in low-income and middle-income countries (LMICs).^1^ In Kenya, NCDs caused almost one-third of all deaths in 2015, and this proportion is likely to rise over half in the coming 10 years. NCDs contribute to a considerable disease burden, accounting for over half of all adult hospital admissions and in-hospital deaths.^2^

There is a good evidence of the cost-effectiveness of prevention and treatment strategies for hypertension and diabetes,^3^ but their implementation remains a challenge. In Kenya, levels of hypertension and diabetes control have remained low.^4 5^ Strengthening primary healthcare for people with NCDs has thus become a priority, with innovative approaches to increase availability of screening, early detection and appropriate management of NCDs such as hypertension being implemented.^6 7^ One such approach has been the inclusion of NCD management in existing primary healthcare platforms that have strong infrastructure and experience in the management of other chronic conditions, such as HIV.^8^ An example is the ‘Academic Model Providing Access to Health Care’ (AMPATH) in western Kenya, one of sub-Saharan Africa’s largest HIV treatment and control programmes, which was progressively expanded to provide NCD care from 2010 onwards.^9^

AMPATH provides the platform for the Primary Health Integrated Care Project for Chronic Conditions (PIC4C).^10^ In 2018, by the Kenyan Ministry of Health in partnership with AMPATH/Moi University, Access Accelerated and the World Bank, PIC4C aims to strengthen primary healthcare services for the prevention and control of NCDs (including hypertension and diabetes) in two counties in western Kenya (Busia and Trans Nzoia) (box 1).

Box 1 **PIC4C activities and implementation**

PIC4C includes screening, early detection of people with hypertension and diabetes, structured referral to different service providers; strengthening of treatment by using structured treatment protocols, training of health workers and community support; improving sustainability health financing by linking patients in care with the voluntary ‘supa cover’ insurance package operated by the National Health Insurance Fund (NHIF) and strengthening of monitoring and evaluation supported by a health information system.

In practice, sites that implemented PIC4C had seven key interventions including (1) Revolving Fund Pharmacies, (2) group cares (where the clinician would meet patients in a group and do the usual clinical monitoring activities), (3) patient support group (focused on financial empowerment through income generating activities and NHIF), (4) training, (5) equipment, (6) mentorship and (7) data strengthening.^13^ Online supplemental table S1 contains more information on implementation activities.

PIC4C, Primary Health Integrated Care for Chronic Conditions.

Emerging evidence suggests that integration of HIV and NCD care is feasible and can be effective in, for example, improving the identification of undiagnosed NCDs or reducing duplication and fragmentation of services.^10^ However, there remains uncertainty about the effects of these activities on clinical outcomes. Also, there are concerns whether integration might have a negative impact on the quality of care achieved by HIV programmes.^11^ This study seeks to contribute to the emerging evidence on integrating primary healthcare in LMIC by reporting on the health benefits and potential unintended consequences of the implementation of the PIC4C model of care in western Kenya, specifically the association of the implementation of PIC4C on people with hypertension, diabetes and/or HIV. Specifically, our objectives were to evaluate the association of the PIC4C programme with the recruitment of new patients and with their change in blood pressure and glucose levels.

## Methods

### Principal study design and setting

The analyses presented in this paper are part of a larger mixed-methods study (PIC4C Scale Up Study) to understand how well PIC4C delivers on its intended aims and to inform and support scale-up of the PIC4C model for integrated NCD management in Kenya. In this paper, we report the quantitative component of the study, the qualitative results will be reported separately, full details of the PIC4C Scale Up Study can be found in the published protocol.^12^

Specifically, we conducted a retrospective cohort study using data from the AMPATH Medical Records System (AMRS).

The study setting includes eligible facilities in Busia and Trans Nzoia counties operating with AMRS and including management of people with NCDs for at least 2 years prior to PIC4C implementation. The 30 clinics included in this analysis were all previously sites for chronic disease management (CDM) supported by AMPATH through previous grants. These CDM clinics had existing treatment for hypertension and diabetes, among other NCDs with HIV clinics that were running parallel in the same facility but in different locations. The integration of care was for chronic non-HIV care. At the time of commencement of the project none of the facilities or neighbouring communities had any ongoing screening for hypertension or diabetes. The only other primary care access to these services is existing private for-profit clinics, which are often run by individuals and situated in town centres. Additionally, most staff managing the conditions had not been offered additional training or any mentorship on the two conditions but were using the knowledge gained from their formative college training.

Drug stock rates at the onset of the project were less than 40% as per a baseline survey conducted, and most patients had to buy medicines in the local private chemists. There were MoH data collection tools, but these were not available nor used by the facilities and hence patients used booklets which they carried home.^13^

The prevalence of hypertension for adults in Kenya as per Stepwise survey 2015 was 24.5%, and the catchment population for the 30 clinics is about 400 000 adults.^14^

The intervention started with a strong community entry, done together with the county and local leadership after signing of the MoU. This was done through several community barazas in which the proposed project was discussed, community views taken and questions answered.

### Inclusion and exclusion criteria

We included 30 PIC4C facilities across Busia and Trans Nzoia counties that had data on blood pressure and plasma glucose levels prior to PIC4C implementation; of these, 28 facilities had data on viral load with a wide variation between facilities, from 1 to 38 970 visits. Further to that, 14 had data on less than 30 visits and fewer than 8 patients over the study period (online supplemental table S2). To minimise sparse data bias, these were excluded from analysis.^15^ This left us with 14 facilities with data on viral load. We excluded patient data with a systolic blood pressure (SBP) below 50 mm Hg or over 250 mm Hg and with a diastolic blood pressure (DBP) below 40 mm Hg or over 120 mm Hg assuming data entry errors. In each facility, we included all adults aged over 18 years with a diagnosis of hypertension, diabetes and/or HIV/AIDS.

### Sample size

Prior to the study, and assuming a sample size of 8000 patients with hypertension and 1000 with diabetes, we estimated that we would have over 90% power to detect a reasonable and relevant association of the PIC4C for our primary outcomes, hypertension and diabetes, on the assumption of the simplest set-up of a before-and-after study, where the power calculation is based on a paired t-test with the following corresponding assumptions: SBP change of 5 mm Hg (SD 15) intraclass correlation coefficient (ICC) 0.05, haemoglobin A1C (HbA1c) change 0.37% (SD 1.1%) ICC 0.04.^16^ Given our more complex design, this sample size calculation demonstrates that our study was well powered to detect a relevant and feasible effect for hypertension and diabetes. We did not estimate sample size using power calculations for people living with HIV that are virally suppressed and for service utilisation (ie, the number of new patients) as these were considered secondary outcomes. Further details can be found in the protocol.^11^ We generated descriptive statistics using patient-level and visit-level data for patients with hypertension, diabetes and HIV.

### Intervention implementation index

The implementation of PIC4C was not uniform across times and facilities and there was not a simple ‘cut-off’ point before and after the implementation. Therefore, we generated a ‘PIC4C implementation index’ to account for spatial and temporal differences between facilities in implementing the seven key PIC4C intervention components: Revolving Fund Pharmacies (RFPs), group cares, patient support group, training, equipment, mentorship and data strengthening. While most facilities implemented intervention components between June 2019 and November 2019, some facilities had implemented at least one activity as early as January 2017, progressively adding further components over time. 10 of the 30 included facilities had implemented all seven activities by November 2019 and throughout the study period to December 2021. The index was calculated for each facility as a continuous variable with a score from 0 (no implementation) to 1 (full implementation) to reflect the intensity of the intervention (number of components) and the extent to which the seven intervention components were implemented (length of implementation).

online supplemental file 2 shows the PIC4C implementation index indicating that at least one activity was implemented as early as January 2017 with a scale-up of activities between April and November 2019.

We generated two indices: (a) using equal weights for each implementation component, used for primary analysis and (b) a weighted score for sensitivity analysis; this was informed by discussions with key stakeholders (PIC4C staff), and we assigned the following weights: 20% for RFPs, group cares and patient support group; 10% for training, equipment, mentorship and data strengthening.

### Definitions

#### Hypertension

We defined a patient with hypertension as someone who, at any visit, had an SBP≥160 mm Hg or DBP≥100 mm Hg; took hypertension medication (amlodipine, atenolol, enalapril, felodipine, hydrochlorothiazide, lisinopril, losartan, metoprolol, nifedipine, telmisartan); was prescribed but missed hypertension medication (at a given visit) or had an SBP≥140 mm Hg or DBP≥90 mm Hg over two consecutive visits.

#### Diabetes

A patient with diabetes was defined as someone who, at any visit, had a random plasma glucose (RPG) ≥200 mg/dL (11.1 mmol/L) and/or fasting plasma glucose (FPG) ≥126 mg/dL (7.0 mmol/L); took diabetes medication (glibenclamide, gliclazide, glimepiride, neutral protamine Hagedorn (NPH) insulin, NPH insulin 70%–30%, metformin) or was prescribed and missed diabetes medication (at a given visit).

#### Time since diagnosis of hypertension and diabetes

This was defined using self-reported data or using the first visit recorded during the study period where self-reported data were missing.

#### Viral suppression

A patient with a viral load below 1000 copies/mL was defined as virally suppressed.

#### New patient

A new patient was defined as a patient who was seen for the first time since January 2017 or who was seen again following a gap between visits of at least 6 months.

### Data sources

We conducted aggregate (facility) level and individual (patient) level analysis to assess service utilisation and clinical effectiveness, respectively. For both analyses, we used the AMRS that integrates data from patients with diabetes, hypertension and HIV health record systems.

### Analysis

#### Service utilisation

To assess service utilisation, we sought to estimate the monthly proportion of new patients seen at the facilities before and after PIC4C implementation.

#### Design and analysis

We used a multilocation single group interrupted time series design (ie, health services (facility) panel-level data with a monthly temporal resolution and no controls) for the period July 2017 to December 2021 with November 2019 defined as the PIC4C implementation cut-off time point.

We used segmented binomial regression, which divides the time series into preimplementation and post-implementation segments using November 2019 as the cut-off, with random effects for facilities to estimate the change in the monthly proportion of new patients before and after PIC4C implementation. We calculated unadjusted models and models adjusted for sex (% of patients who are female), age and association of COVID-19 policies. Monthly time and autoregressive of order 1 (to account for serial autocorrelation) were included in both unadjusted and adjusted models.

#### Clinical effectiveness

We used individual (patient visit) level data to assess the association of PIC4C with outcomes for hypertension, diabetes and viral suppression for the same period.

#### Outcomes

Primary outcome measures were SBP and FPG (continuous variables). Secondary outcome measures included DBP, hypertension control (having SBP/DBP<140/90 mmHg), random plasma glucose, diabetes control (having plasma glucose FPG<7.0 mmol/L or RPG<11.1 mmol/L), viral load (copies/ml) and HIV viral suppression (viral load <1000 copies/mL).

#### Covariates

We included sex, age, time in the AMPATH programme, the proportion of new patients with hypertension and/or diabetes per month, and comorbidity (ie, patients with both hypertension and diabetes) in our models, as appropriate (see Design and analysis section). We generated a service colocation index to identify whether NCD and HIV services were located together or not in different facilities.

The postimplementation period of PIC4C from November 2019 coincided with the COVID-19 pandemic and we sought to account for the association of the pandemic using the ‘COVID-19 Government Response Stringency Index’ developed by researchers at Oxford University.^17^ Following the confirmation of the first Covid case on 12 March 2020, Kenya progressively introduced a range of measures, with the first strict lockdown implemented by the end of March 2020.^18^ The Covid Stringency Index quantifies pandemic-related containment and closure (‘lockdown’) policies on a daily basis for over 180 countries. For this analysis, we used the ‘index’ reported in the last day of each month to generate a proxy monthly measure for the association of COVID-19 on restricting people’s mobility (eg, accessing health facilities).

#### Design and analysis

We used generalised mixed-effects linear models (fixed effects for facilities and random effects for patients) with a binomial distribution for binary outcomes (hypertension and diabetes control and viral suppression) and Gaussian distribution for continuous measures (SBP, DBP, RPG, FPG and viral load) using meglm in Stata (StataCorp. 2021. *Stata Statistical Software: Release 17*. College Station, TX: StataCorp LLC.). We calculated unadjusted and adjusted models. Variables adjusted for included, sex, age, hypertension (or diabetes), time in the programme, time since hypertension (or diabetes) diagnosis, services colocation (for hypertension and diabetes), proportion of new patients with hypertension or diabetes per month, association of COVID-19 policies and seasonality (hypertension only). For people living with HIV, we adjusted for sex, age, comorbidity (diabetes, hypertension or both), time in the programme, proportion of new patients with hypertension and/or diabetes per month and association of COVID-19 policies.

In our primary analysis, we used the equal weighted PIC4C implementation index and data from all sites and all visits to examine the effects of the PIC4C implementation index and the individual PIC4C implementation components on our primary and secondary outcomes. In sensitivity analysis, we restricted analysis to the third recorded visit onwards (as we assumed that at least two visits are required before a patient would have their blood pressure and plasma glucose levels controlled) and restricted to PIC4C sites that had implemented all seven activities. We also examined the association of the unequal weights PIC4C implementation index on our primary and secondary outcomes. Results are reported as OR for binary outcomes interpreted as the OR for a full implementation (score 1) vs no implementation (score 0) and beta coefficients (for continuous outcomes) with 95% CIs. We did not perform imputation of missing values and performed a complete case analysis using data on age, sex, blood pressure and diabetes measurements and viral load.

### Weighting

The intervention ‘PIC4C’ implementation index comprises seven implementation activities, as described in box 1. The weighting was applied to account for the intensity and length of the intervention at a given facility (ie, temporal differences) as well as capture the fact that not all facilities implemented PIC4C at the same time (ie, spatial differences).

Specifically, we applied a weight for each of the seven implementation activities to account for both the total number of activities that were implemented at a given time point and how long these were implemented. For the index with equal weights, we assumed that each of the seven implementation activities would exhibit the same intervention intensity and each activity was given a weight of 1/7 or 0.14, that is, dividing a score of 1 for all seven activities by the total number of activities.

At any given point in time, a facility would have had no activities implemented (and therefore a score of 0 for the index at a specific point in time), one activity (a score of 0.14 for the index at a specific point in time and thereafter), more activities (a score of 0.29 or more for the index at a specific point in time and thereafter) and all seven activities (a score of 1 for the index at a specific point in time and thereafter). In online supplemental file 3, we describe an example of a facility and how the index with equal weights is calculated and captured over the course of the study.

In the sensitivity analysis, we used unequal weights following discussions with key stakeholders under the assumption that some implementation activities had a greater intensity than other activities. In online supplemental table S3, we present the weights given for each activity for the index with the equal and unequal weights.

## Results

We analysed data from 66 641 visits of 13 046 patients with hypertension and 24 005 visits of 7267 patients with diabetes attending 30 PIC4C facilities. We also analysed data from 84 855 visits of 21 186 people living with HIV from 14 PIC4C facilities (table 1).

**Table 1:** Patient-level and visit-level characteristics for the three patient populations

Patient-level characteristics	Patients with hypertension (n=13 046)		Patients with diabetes (n=7267)		Patients with HIV (n=21 186)	
	Np	%	Np	%	Np	%
Sex						
Female	9296	71.3	4753	65.4	14 543	68.6
Male	3750	28.7	2514	34.6	6643	31.4
Age group						
18–44	2143	16.4	1341	18.5	13 061	61.6
45–64	6066	46.5	3456	47.6	7552	35.6
65+	4837	37.1	2470	34.0	573	2.7
Time since diagnosis						
Less than a year	10 174	78.0	5511	75.8	-	-
1–5 years	2270	17.4	1421	19.6	-	-
6–10 years	332	2.5	189	2.6	-	-
More than 10 years	270	2.1	146	2.0	-	-
Total number of visits						
1 visit	4352	33.4	3725	51.3	2750	13.0
2–5	4783	36.7	2280	31.4	14 795	69.8
6–10	1993	15.3	757	10.4	3637	17.2
10+	1918	14.7	505	6.9	4	0.0
Comorbidity						
DM	2068	15.9				
HTN			5636	77.6		
HTN and DM					262	1.2
DM					223	1.1
HTN					10	<1
Visit-level characteristics	Nv	Mean (SD)	Nv	Mean (SD)	Nv	Mean (SD)
Systolic blood pressure (mm Hg)	66 622	144.8 (21.9)				
Diastolic blood pressure (mm Hg)	66 622	85.0 (13.1)				
Random plasma glucose (mmol/L)			15 778	10.7 (5.8)		
Fasting plasma glucose (mmol/L)			8268	8.8 (4.6)		
Viral load (copies/mL)					84 855	3896 (33944.8)
Total time in system (days)	66 622	888 (551.6)	24 005	857 (561.2)	84 855	1042 (360.9)
Time between visits (days)	53 705	95 (115.1)	18 557	95 (120.0)	63 669	289 (139.7)

DMdiabetes mellitusHTNhypertensionNpnumber of patientsNvnumber of visits

Visits from patients with hypertension were, on average, more than twice the number of visits from patients with diabetes. However, patterns were similar with a peak in monthly visits in mid-2019 when most of the PIC4C activities were being implemented. The number of monthly clinic visits fell in 2020 and in 2021, most likely because of the COVID-19 pandemic (online supplemental file 4). We observed a small increase in the monthly mean age of patients with more females, compared with males, attending clinics in the postimplementation period (onlinesupplemental files 5^8^).

Patients with hypertension and diabetes were predominately female and aged 45 years or more. People living with HIV were predominately female and younger than 45 years. Most patients with hypertension and diabetes had been diagnosed within the past year; about half of patients with diabetes had one clinic visit and over one-third with hypertension had between 2 and 5 visits over the study period with an average time of 95 days between visits. Approximately one in seven patients with hypertension also had diabetes, whereas more than three-quarter of patients with diabetes also had hypertension. The proportion of people living with HIV diagnosed with either hypertension or diabetes was very low (table 1).

### Service utilisation

**Figure 1:**
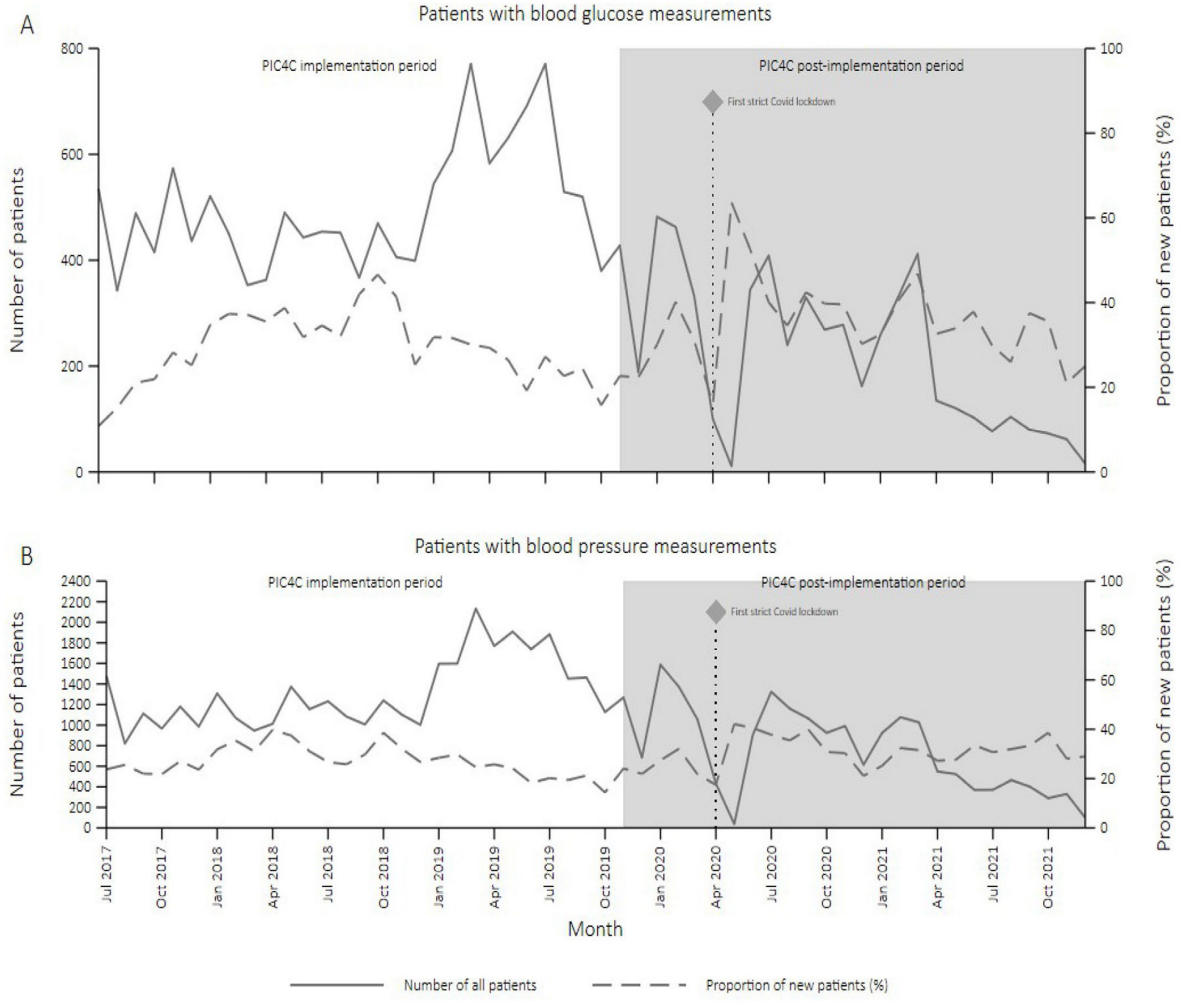
Change in the proportion of new patients with (A) hypertension and (B) diabetes visiting PIC4C clinics between January 2017 and December 2021. PIC4C, Primary Health Integrated Care for Chronic Conditions.

There was evidence of an increase in the proportion of new patients with hypertension and/or diabetes per month between the PIC4C preimplementation (January 2017–November 2019) and postimplementation periods (December 2019–December 2021) (table 2, figure 1online supplemental files 9; 10).

**Table 2:** Segmented generalised linear regression models of the change in the proportion of new patients with hypertension and diabetes before and after PIC4C implementation

	Hypertension		Diabetes	
	OR (95% CI)	aOR (95% CI)*	OR (95% CI)	aOR (95% CI)*
Step change	1.53 (1.36 to 1.73)	1.57 (1.39 to 1.78)	1.32 (1.20 to 1.45)	1.31 (1.19 to 1.45)
Sex (% females)		1.03 (0.78 to 1.35)		0.85 (0.66 to 1.09)
Age		0.99 (0.98 to 1.00)		0.98 (0.97 to 0.99)
Covid index		0.91 (0.79 to 1.04)		0.96 (0.86 to 1.08)

* Models adjusted for sex (% of patients thatwho are female), age, association of covidCOVID-19 policies that primarily restrict people’s behaviour and monthly time points. A new patient was defined as a patient who was seen for the first time since January 2017 or who is seen again following a gap between visits of at least 6 months.

aORadjusted OR

### Clinical effectiveness

In the primary analyses, we found that implementation of the PIC4C project was associated with small increases in SBP (1.7 (95% CI 0.8 to 2.7) mm Hg) and FPG (0.6 (95% CI 0.0 to 1.1) mmol/L (table 3). We also found evidence of an association between the PIC4C implementation and a decrease of the proportion of patients who had their blood pressure and plasma glucose levels under control (adjusted OR and 95% CI 0.77 (0.68 to 0.88) and 0.59 (95% CI 0.47 to 0.73), respectively), and an increase in DBP (aBeta 1.20, 95% CI 0.62 to 1.77). We did not find evidence of an association between PIC4C implementation and RPG (aBeta 0.46, 95% CI −0.13 to 1.05) (online supplemental table 4A,B).

**Table 3:** Association between PIC4C implementation and primary outcomes (systolic blood pressure and fasting plasma glucose)

	SBP (mm Hg)		FPG (mmol/L)	
All visits and sites (SBP: n_v_=66 641; FPG: n_v_=8268)	Beta (95% CI)	aBeta (95% CI)*	Beta (95% CI)	aBeta (95% CI)*
PIC4C implementation index	**9.64 (8.83 to10.44**)	1.73 (0.75 to 2.71)	**1.48 (1.07 to1.89**)	0.55 (0.01 to 1.08)
Sex (reference: female)				
Male		**1.81 (1.16 to2.45**)		−0.10 (−0.41 to 0.22)
Age group (reference 18–44 years)				
45–64		1.68 (0.90 to 2.46)		**−0.58 (−0.96 to-0.20**)
65+		**5.41 (4.59 to6.24**)		**−1.16 (−1.57 to-0.74**)
Comorbidity				
DM (reference: no DM)		**−4.98 (−7.88 to−2.08**)		
HTN (reference: no HTN)				**−0.51 (−0.85 to−0.17**)
Years since diagnosis		0.07 (−0.01 to 0.15)		**0.08 (0.05 to0.12**)
Time in the programme		**−3.08 (−3.31 to−2.84**)		**−0.32 (−0.45 to−0.19**)
Colocation of NCD/HIV services (Reference: located separately)				
Colocated		−1.43 (−2.94 to 0.09)		−0.29 (−1.09 to 0.50)
Covid Stringency Index		**5.79 (4.97 to6.61**)		0.57 (0.07 to 1.06)
Proportion of new HTN or DM patients per month		0.93 (−0.37 to 2.24)		**−0.93 (−1.74 to−0.13**)
Sensitivity analysisRestricted to sites that implemented all seven activities and excluding first two visits (n_s_=10; SBP: n_v_=9927; FPG: n_v_=1610)	Beta (95% CI)	aBeta (95% CI)*	Beta (95% CI)	aBeta (95% CI)*
PIC4C implementation index	**5.70 (3.91 to7.49**)	1.74 (−0.70 to 4.17)	**1.93 (1.03 to2.82**)	0.52 (−0.64 to 1.67)
Sex				
Male (reference: female)		0.46 (−1.56 to 2.47)		−0.43 (−1.23 to 0.38)
Age group				
45–64 (reference 18–44 years)		−1.08 (−3.44 to 1.27)		−0.94 (−1.89 to 0.01)
65+		1.60 (−0.88 to 4.08)		**−1.78 (−2.79 to-0.77**)
Comorbidity				
DM (reference: no DM)		−26.35 (−65.92 to 13.22)		
HTN (reference: no HTN)				−0.28 (−1.15 to 0.59)
Years since diagnosis		0.21 (−0.05 to 0.47)		**0.15 (0.06 to0.25**)
Time in the programme		**−0.75 (−1.44 to−0.06**)		**−0.75 (−1.08 to−0.42**)
Colocation of NCD/HIV services				
Colocated (reference: located separately)		5.42 (0.80 to 10.03)		3.28 (−1.39 to 7.94)
Covid index		**4.31 (2.33 to6.30**)		0.55 (−0.51 to 1.60)
Proportion of new HTN or DM patients per month		−0.03 (−3.94 to 3.87)		**−2.94 (−5.17 to−0.70**)

p<0.05 highlighted in bold.

* Mixed effects models using random effects for patients and fixed effects for sites.

aBetaadjusted beta coefficientDMdiabetes mellitusFPGfasting plasma glucoseHTNhypertensionNCDnon-communicable diseasen_s_number of sitesn_v_number of visitsPIC4CPrimary Health Integrated Care for Chronic ConditionsSBPsystolic blood pressure

Being male, over the age of 65 years and COVID-19 restrictions were all associated with higher SBP (table 3). Hypertension and diabetes comorbidity and time in the AMPATH programme were associated with lower SBP (table 3).

We found evidence that years since diagnosis and COVID-19 restrictions were associated with higher FPG. Conversely, being over 65 years, having both hypertension and diabetes, time in the programme, and the proportion of new diabetes patients were all associated with lower FPG (table 3). We found similar trends for other clinical outcomes for diabetes and hypertension. PIC4C implementation was further associated with a decline in the proportion of patients who had their blood pressure and plasma glucose levels under control, and an increase in DBP (online supplemental table S4A,B). We did not find strong evidence of an association between PIC4C implementation and RPG (online supplemental table S4B).

### Sensitivity analyses

There were 10 sites that implemented all 7 PIC4C activities. After restricting analyses to data from the third clinic visit onwards at these sites (n=9927 visits or hypertension and n=1610 visits for diabetes), we did not find strong evidence of an association between PIC4C implementation and SBP or with FPG (table 3, online supplemental table S5). When looking at individual implementation activities, we found some association between RFPs and SBP, and some association all components except equipment and FPG (online supplemental table S6)

Looking at individual PIC4C activities, we found some evidence for a positive association between RFPs, mentorship and patient support group and SBP, whereas training was associated with a lower SBP (table 4). We also found evidence that training was associated with an increase, whereas RFPs and patient support group were associated with a decrease in the proportion of patients who had their blood pressure under control (online supplemental table S7).

**Table 4:** Association between individual PIC4C implementation components and systolic blood pressure (SBP) and fasting plasma glucose (FPG)

	SBP	FPG
	aBeta (95% CI)*	aBeta (95% CI)†
Training	**−0.56 (−1.01 to −0.10)**	0.12 (−0.14 to 0.38)
RFPs	**2.72 (2.12 to 3.33)**	**0.51 (0.18 to 0.84)**
Mentorship	**0.52 (0.07 to 0.98)**	0.13 (−0.15 to 0.41)
Data strengthening	0.3 (−0.22 to 0.82)	0.13 (−0.14 to 0.41)
Patient support group	**1.74 (1.13 to 2.35)**	**0.56 (0.23 to 0.90)**
Equipment	−1.29 (−4.80 to 2.22)	2.3 (−1.84 to 6.45)
Group cares	−0.19 (−1.05 to 0.67)	−0.47 (−1.04 to 0.11)
Sensitivity analysis: restricted to sites that implemented all seven activities (n=10) and excluding first two visits		
Training	−1.15 (−2.37 to 0.07)	−0.22 (−0.78 to 0.34)
RFPs	2.1 (0.72 to 3.48)	0.75 (0.02 to 1.49)
Mentorship	−0.1 (−1.40 to 1.20)	0.09 (−0.56 to 0.74)
Data strengthening	0.8 (−0.49 to 2.09)	0.21 (−0.36 to 0.79)
Patient support group	**2.92 (1.37 to 4.47)**	0.72 (−0.01 to 1.46)
Equipment‡	-	-
Group cares	0.81 (−2.44 to 4.07)	−1.58 (−4.50 to 1.35)

p<0.05 highlighted in bold

* Mixed effects models using random effects for patients and fixed effects for sites adjusted for sex, age, diabetes, time in the programme, time since hypertension diagnosis, services co-location, association of covid policies that primarily restrict people’s behaviour, proportion of new patients with hypertension per month and seasonality.

† Mixed effects models using random effects for patients and fixed effects for sites adjusted for sex, age, hypertension, time in the programme, time since diabetes diagnosis, services co-location, association of covidCOVID-19 policies that primarily restrict people’s behaviour, and proportion of new patients with diabetes per month.

‡ All facilities had equipment as of January 2017.

aBetaadjusted betaPIC4CPrimary Health Integrated Care for Chronic ConditionsRFPsRevolving Fund Pharmacies

Equipment and group cares were associated with an increase of the proportion of patients who had their blood glucose under control, whereas all other activities were associated with a decrease in diabetes control (online supplemental table S7).

Finally, we found no evidence of an association of PIC4C implementation and viral load or viral suppression among people living with HIV (online supplemental table S8). This was still evident even when unequal weights were applied (online supplemental table S9)

## Discussion

We examined the health benefits for people with hypertension and diabetes and potential unintended consequences for HIV viral of the implementation of the PIC4C model in western Kenya. We found evidence that the implementation of the PIC4C led to an increase in the proportion of new patients with diabetes and hypertension attending clinics. Conversely, in terms of clinical outcomes, we found that following PIC4C implementation small increases in both SBP and FPG were compatible with our data. However, when we restricted the analysis to facilities that had fully implemented all seven PIC4C components (and considering the third visit onward) the evidence for these associations got weaker and both increases and decreases in SBP and FPG were compatible with our data. Furthermore, exploration of specific PIC4C components pointed to differential associations, with equipment associated with decreases in SBP and training associated with an increase in blood pressure control. Concerning outcomes for people living with HIV, we did not find strong evidence that PIC4C implementation was associated with an increase in viral load, which is an important aspect when considering implementing NCDs programmes in mature healthcare services which were developed mainly for HIV (like AMPATH).

McCombe *et al*^10^ reviewed the evidence of integrating NCD care with HIV care in sub-Saharan Africa and found that, in general, related approaches are potentially feasible and effective; however, the majority of reviewed studies were small-scale and did not focus on clinical effectiveness, limiting generalisability of findings. Similar to evidence reported by McCombe *et al*, our study found that the PIC4C model has been successful in the identification of previously undiagnosed NCDs. Yet, we did not find strong evidence that the model led to improvements in clinical outcomes for people with hypertension or diabetes. These apparent ‘negative’ findings need to be interpreted in the context within which the PIC4C model was implemented. First, as a complex intervention which includes multiple components that were implemented over an extended period, it is possible that we did not capture the entirety of the ‘intervention’ in all its dimensions. Second, as noted PIC4C, was successful in identifying previously undiagnosed NCD patients and it is likely that these ‘new’ patients had a worse clinical profile, which we were unable to fully adjust for. Thus, our crude analysis of the association between PIC4C implementation and clinical outcomes found evidence of a positive association which was however attenuated substantially on inclusion of potential cofounders in the adjusted analysis. For example, the crude analysis found that PIC4C was associated with an increase in SBP of 9.6 mm Hg, but this fell to only 1.7 mm Hg in the adjusted analysis. Third, we used secondary data from an electronic health records database, which is subject to known quality limitations as it relates to data completeness, and which could have limited our analysis (see Limitations). Fourth, PIC4C implementation coincided with the COVID-19 pandemic. We found evidence that COVID-19 restrictions were associated with an increase in SBP and FPG. This suggests that COVID-19 restrictions affected the patient population differentially, with a potentially sicker patient population attending clinics. Fifth, we were only able to assess associations postimplementation for a period of 2 years (December 2019–2021). Finally, it is possible that a longer follow-up period would have identified stronger evidence of association as reported in other studies evaluating the long-term effects of integrated care models.^19 20^

### Strengths

To the best of our knowledge, this is the largest study using electronic health records in sub-Saharan Africa that examined the associations with hypertension and diabetes control for a healthcare model that integrated NCD services into an existing primary care HIV platform. We included data from both NCDs and HIV patients, and in our analysis, we included both clinical (change in blood pressure, glucose, viral load) and healthcare service (new patients) outcomes in our analysis.

### Limitations

We intended to use directed acyclic graphs to examine the association with potential confounders and effect modifiers, such as smoking, alcohol and salt intake, body mass index, Antiretroviral Therapy (ART) regimen and time on ART on hypertension, diabetes and viral suppression and inform our models. However, we were either not able to collect information or had missing data on these variables, so we were unable to conduct this analysis. It is thus possible that there was residual confounding in our results.^1421^,^25^ This might be particularly important in the case of the COVID-19 restrictions that coincided with the postimplementation period of PIC4C, and which have likely shaped the nature of patients attending the clinics, specifically, more complex patients with higher SBP or FPG. The COVID-19 stringency index only captures restrictions imposed to people’s mobility at a national level and we were unable to adjust for possible regional variation in the two counties that implemented PIC4C. Sicker patients tend to be referred to facilities which have mentors (often more senior/experienced clinicians) and these are the same facilities that tend to have RFPs due to their broader drug formulary. This could have introduced a selection bias and may be the reason for the negative association of RFP and proportion of patients with controlled blood pressure. Furthermore, our study design, that is, a cohort study without a control group, is also an important limitation to permit any strong conclusion about causality. Finally, another limitation is the lack of information on potential differences between the selected facilities.

#### Implications for research

This study was able to draw on a rich data set derived from electronic health records, which have become increasingly important for the conduct of real-world effectiveness studies to support decision-making where long-term outcomes or subgroup effect estimates are unavailable from randomised controlled trials.^26^ Furthermore, use of electronic health records can help build and support learning health systems and evaluate complex interventions and real-life implementation of models of care.^27 28^ The increasing availability of electronic health records in a wider number of countries and regions such as sub-Saharan Africa opens the possibility of this important research area. There is a need to explore and develop innovative methodological approaches to overcome some of the limitations, such as data quality and completeness, to maximise the output from this rich source of data in LMICs. We have created one of the largest electronic health record cohort of patients with hypertension and/or diabetes in sub-Saharan Africa and we will keep exploring some of these methodological challenges and invite other research groups to collaborate and use this large dataset to improve this important methodological area.

It is also important to triangulate our quantitative analysis with other research approaches. As noted, this study is part of a larger project (scale-up PIC4C), which also includes qualitative research to better understand the implementation process of PIC4C from the perspective of the healthcare providers and decision-makers and to understand patients’ experiences of the care model.^12^ Integrating the findings of the quantitative and qualitative research streams will be important to set our findings in the wider context of PIC4C implementation.^29^ Future research should look at the long-term effects of the PIC4C implementation given what we know about the time it takes to embed novel service approaches into routine settings.^30^

#### Implications for practice

Although we conducted a very thorough and complex analysis, the nature of our quasi-experimental study design and the limited variables that we were able to include in our analysis preclude formulating clear conclusions about the association between PIC4C implementation and clinical outcomes. Having said that, our study found clear evidence that continuous community-based screening for hypertension and diabetes is important for institutionalising early case finding. However, this must go hand in hand with equipping primary care health facilities and training of healthcare workers for any meaningful clinical outcomes to be realised. Our qualitative work as part of the PIC4C Scale Up project might provide further insight into the specific components of the PIC4C care model, how they work and why (or why not). Going forward, it will be important to work with key decision-makers at both regional and national levels to understand and interpret findings to inform future steps regarding the scale-up of different components of PIC4C.^12^

## Conclusion

We found evidence that PIC4C implementation was associated with an increase in new patients with hypertension and diabetes attending clinics and that the implementation of some components, such as training and equipment, were associated with a decrease on SBP and diabetes control. However, we did not find strong evidence of an association between PIC4C implementation and improvement on clinical outcomes and, in fact, a small deterioration on hypertension and diabetes control are compatible with our data. The fact that the immediate post-PIC4C implementation period coincided with the COVID-19 pandemic is likely to explain some of our findings. Future research looking into the long term of PIC4C, triangulation with our ongoing qualitative research and further discussion with decision-makers will further inform the scale-up of PIC4C beyond the study sites.

## supplementary material

10.1136/bmjph-2023-000146 online supplemental file 1

10.1136/bmjph-2023-000146 online supplemental file 2

10.1136/bmjph-2023-000146 online supplemental file 3

10.1136/bmjph-2023-000146 online supplemental file 4

10.1136/bmjph-2023-000146 online supplemental file 5

10.1136/bmjph-2023-000146 online supplemental file 6

10.1136/bmjph-2023-000146 online supplemental file 7

10.1136/bmjph-2023-000146 online supplemental file 8

10.1136/bmjph-2023-000146 online supplemental file 9

10.1136/bmjph-2023-000146 online supplemental file 10

## Data Availability

Data are available on reasonable request.
