# Supplementary material for: Evaluating the implementation of the Primary Health Integrated Care Project for Chronic Conditions: a cohort study from Kenya
Source: BMJ Public Health. 2024 Mar 25;2(1):e000146. doi: 10.1136/bmjph-2023-000146 (PMC7616119; doi:10.1136/bmjph-2023-000146)
Supplement: online supplemental file 1 [file bmjph-2-1-s001.pdf]

Table S1: PIC4C Implementation activities

Activity	Strategy	Personnel	Inputs	Outputs	Outcome
Screening	Community mobile screening booths	Community health promoters (CHP) and CHV	-CHV/CHP trainings<br>-Screening equipment<br>-Mobile phones for data collection<br>-Data bundles<br>-Manual national registers<br>-Transport facilitation supervisors/mentor time effort	Number of people educated, screened, and referred<br>Number of those referred who linked to care<br>CHV/CHP retention rates<br>Evaluation data	-Change in community knowledge<br>-Change in screening rates and Linkage rates<br>-Data for informed decision making/planning
	Facility triage site screening	CHP<br>Nurses<br>HRIOs	Training<br>Equipment<br>Mobile tablets for data collection<br>Manual Registers<br>Screening trainings	Number screened<br>Number referred to clinic after diagnosis	Change in triage screening rates<br>Change in practice
HCWs training	Classroom structured training and online CPDs	Nurses<br>Clinical officers<br>Pharmaceutical techs<br>Health record Information Officers (HRIOs)<br>Medical officers<br>Nutritionists	Trainers<br>Training curriculum<br>Training facilitation<br>Information and education materials<br>Internet bundles for CPDs	Number of trainings and online CPDs<br>Number of health workers trained<br>Topics covered in trainings	Change in knowledge measured by pre and post test<br>Change in attitude assessed through baseline and End-line Likert scale test
	On job mentorship	Nurses<br>Clinical officers<br>Pharmaceutical techs<br>Health record Information Officers (HRIOs)<br>Medical officers<br>Nutritionists	County based clinical mentors time effort<br>Mentorship transport<br>Mentors training	Mentorship reports<br>Support supervision reports	Improved diagnostic and management decision making skills measured through process evaluation
Supply chain	Revolving Fund Pharmacies	Pharmacists<br>Pharmaceutical technologists<br>Cashiers<br>Dispensary nurses	Space renovation and set up<br>MOU signing meetings with County officials and community reps<br>Seed stock of drugs<br>Computer/tablets for data capture<br>Transport and facilitation for supervision and mentorship	Number of prescription served<br>Financial reports<br>Stock rates	Increased availability of medication<br><br>Reduced cost of accessing medication<br>As per final qualitative evaluation

Patient management and follow up	Treatment in integrated primary care NCD clinics ((separate from the HIV clinics) at level 4 and 3 facilities by clinical officers, visiting mentor medical officers or consultants<br><br>Treatment in general clinics at level 2 by dispensary nurse or visiting clinical officer mentor	Clinical officers<br>Medical officers<br>Consultant physicians<br>Nurses<br>Health records information officers<br>Nutritionists<br>CHPs for triage and education<br>Lab techs<br><br>Nurses<br>CHPs/CHVs<br>Occassionally Visiting mentor<br>Clinical officers	Equipment such as Omron automatic BP machines, weight meters, height meters, glucose meters, HbA1c machine, Stethoscopes,<br><br>Data tools: Patient files, MoH Registers, Booking cards, and referral cards, Mobile tablets for electronic data capture<br><br>Healthcare workers training and mentorship	Number of new and returning patients treated<br><br>Number of patients given health education<br><br>Number of patients referred upwards for review<br><br>Number of patients referred to support groups	Blood pressure control rates<br><br>Blood glucose control rates<br><br>Adherence rates (self reported)<br><br>Retention rates
Leadership and governance strengthening	Engagement for development and signing of MoU<br><br>Data Review meetings<br><br>Facility board management teams leadership trainings<br><br>Result dissemination meetings	Health facility leadership<br>Sub-county leadership<br>County health leadership<br>County health committee leadership<br>National NCD leadership<br>National health insurance fund local leadership<br>Medical Professional bodies leadership	Meeting facilitation<br><br>Training facilitation	Minutes of meetings<br><br>Memorundums of understanding<br><br>Training attendance lists	Increased adoption of project strategies into mainstream practice
Patient self management support	Patient support groups	Patient peer leaders<br>Patients<br>Community health workers and promoters<br>Nurses<br>Clinical officers<br>Research assistants	Training<br>Equiping with self monitoring equipment eg Bp machines, glucose meters<br>Support group registers	Number of support groups<br>Number of meetings<br>Topics covered in health education<br>Data records	Increased adherence<br>Increased retention<br>Improved treatment outcomes

Table S2: Total number of visits across the study period in 28 PIC4C facilities with data on viral load.

PIC4C site	N of visits	N of patients*
Aboloi Dispensary	142	38
Akichelesit Dispensary	117	17
Angurai Health Centre	2,670	687
Bumala A Health Centre	7,330	1738
Bumala B Health Centre	3,207	845
Changara Dispensary	503	192
Kamolo Dispensary	112	49
Khunyangu Sub County Hospital	1,279	302
Kitale County Hospital	38,895	9925
Malaba Dispensary	1,702	625
Moding Health Centre	24	8
Mukhobola Health Centre	4,919	1359
Port victoria Sub County Hospital	12,820	3195
Saboti Sub County Hospital	5,750	1483
Budalangi dispensary	6	2
Busagwa dispensary	20	4
Endebes health centre	5	1
Ikonzo dispensary	28	6
Kapsara sub-district hospital	16	2
Kwanza health centre	2	2
Matayos health centre	5,409	1378
Matunda health centre	1	1
Nasewa health centre	21	4
Rukala dispensary	6	1
Sikarira dispensary	11	3
Sirimba mission hospital	12	2
Sisenye dispensary	10	2
Sister freda medical centre	3	1
**Total**	**85,020**	**21,872**

*636 patients had visits in two or more different facilities.

Table S3: Weights given for each activity for the index with the equal and unequal weights.

Implementation activity	Equal weight	Unequal weight
Training	0.143	0.1
Equipment	0.143	0.1
RFPS	0.143	0.2
Mentorship	0.143	0.1
Data Strengthening	0.143	0.1
Group Cares	0.143	0.2
Patient Support group	0.143	0.2
Total (Implementation of all activities)	1	1

Table S4a: Impact of PIC4C implementation on diastolic blood pressure and blood pressure control.

	DBP (mm HG)		BP control	
All visits and sites (n_v=66,641)	Beta (95% CI)	Beta (95% CI)[a]	OR (95% CI)	aOR (95% CI)[a]
PIC4C implementation index	**5.48 (5.01 - 5.96)**	**1.20 (0.62 - 1.77)**	**0.31 (0.28 - 0.35)**	**0.77 (0.68 - 0.88)**
Sex				
Male (Reference: Female)		-0.03 (-0.43 - 0.36)		**0.84 (0.78 - 0.90)**
Age group				
45-64 (Reference 18-44 years)		**-1.08 (-1.55 - -0.60)**		0.98 (0.90 - 1.07)
65+		**-4.08 (-4.59 - -3.58)**		**0.85 (0.78 - 0.94)**
Comorbidity				
DM (Reference: no DM)		**-2.01 (-3.76 - -0.27)**		**1.74 (1.15 - 2.64)**
Years since diagnosis		**0.05 (0.00 - 0.10)**		0.99 (0.98 - 1.00)
Time in the programme		**-1.82 (-1.96 - -1.68)**		**1.33 (1.29 - 1.36)**
Co-location of NCD/HIV services				
Co-located (Reference: located separately)		-0.77 (-1.65 - 0.12)		1.13 (0.93 - 1.36)
Covid index		**3.75 (3.27 - 4.23)**		**0.51 (0.45 - 0.57)**
Proportion of new patients per month		**-2.21 (-2.97 - -1.45)**		1.10 (0.92 - 1.31)
Excluding first two visits (n_v=44,901)	Beta (95% CI)	Beta (95% CI)[a]	OR (95% CI)	aOR (95% CI)[a]
PIC4C implementation index	**3.50 (2.95 - 4.05)**	**1.22 (0.57 - 1.86)**	**0.52 (0.46 - 0.59)**	**0.84 (0.72 - 0.98)**
Sex				
Male (Reference: Female)		**-0.70 (-1.19 - -0.22)**		**0.86 (0.78 - 0.94)**
Age group				
45-64 (Reference 18-44 years)		-0.31 (-0.88 - 0.27)		0.96 (0.86 - 1.08)
65+		**-2.82 (-3.43 - -2.21)**		**0.84 (0.74 - 0.94)**
Comorbidity				
DM (Reference: no DM)		0.59 (-4.99 - 6.17)		1.70 (0.49 - 5.92)
Years since diagnosis		**0.08 (0.03 - 0.13)**		**0.98 (0.97 - 0.99)**
Time in the programme		**-0.62 (-0.80 - -0.43)**		**1.12 (1.08 - 1.17)**
Co-location of NCD/HIV services				
Co-located (Reference: located separately)		**-1.28 (-2.21 - -0.34)**		1.05 (0.85 - 1.30)
Covid index		**2.98 (2.47 - 3.49)**		**0.60 (0.53 - 0.68)**
Proportion of new patients per month		**-1.66 (-2.49 - -0.83)**		1.16 (0.95 - 1.43)
Restricted to sites that implemented all seven activities (n_s=10; n_v=13,625)	Beta (95% CI)	aBeta (95% CI)[a]	Beta (95% CI)	Beta (95% CI)[a]
PIC4C implementation index	**5.72 (4.81 - 6.63)**	-0.39 (-1.64 - 0.86)	**0.29 (0.24 - 0.35)**	0.92 (0.69 - 1.23)
Sex				
Male (Reference: Female)		-0.77 (-1.72 - 0.19)		0.86 (0.72 - 1.03)
Age group				
45-64 (Reference 18-44 years)		-0.99 (-2.13 - 0.15)		1.14 (0.92 - 1.43)
65+		**-4.22 (-5.42 - -3.03)**		1.04 (0.83 - 1.31)
Comorbidity				
DM (Reference: no DM)		6.33 (1.36 - 11.31)		0.65 (0.22 - 1.88)
Years since diagnosis		0.12 (-0.02 - 0.25)		1.00 (0.97 - 1.02)
Time in the programme		**-1.67 (-1.99 - -1.35)**		**1.33 (1.25 - 1.42)**
Co-location of NCD/HIV services				
Co-located (Reference: located separately)		**-5.28 (-7.32 - -3.24)**		1.16 (0.77 - 1.73)
Covid index		**5.67 (4.61 - 6.73)**		**0.40 (0.31 - 0.51)**
Proportion of new patients per month		**-2.43 (-4.48 - -0.39)**		1.58 (0.97 - 2.59)
Restricted to sites that implemented all seven activities and excluding first two visits (n_s=10; n_v=9,927)	Beta (95% CI)	aBeta (95% CI)[a]	Beta (95% CI)	Beta (95% CI)[a]
PIC4C implementation index	3.95 (2.88-5.01)	-0.59 (-2.03 - 0.85)	**0.43 (0.34 - 0.55)**	0.93 (0.66 - 1.32)
Sex				
Male (Reference: Female)		-1.08 (-2.29 - 0.13)		0.89 (0.71 - 1.13)
Age group				
45-64 (Reference 18-44 years)		-0.24 (-1.65 - 1.17)		1.00 (0.75 - 1.33)
65+		**-2.66 (-4.15 - -1.18)**		0.92 (0.69 - 1.24)
Comorbidity				
DM (Reference: no DM)		7.88 (-15.64 - 31.39)		1.00 (0.90 - 1.11)
Years since diagnosis		**0.23 (0.08 - 0.39)**		0.98 (0.95 - 1.01)
Time in the programme		**-0.66 (-1.07 - -0.25)**		**1.14 (1.05 - 1.24)**
Co-location of NCD/HIV services				
Co-located (Reference: located separately)		-2.18 (-4.95 - 0.59)		0.88 (0.51 - 1.52)
Covid index		**5.33 (4.16 - 6.50)**		**0.47 (0.35 - 0.64)**
Proportion of new patients per month		**-2.64 (-4.94 - -0.34)**		1.68 (0.95 - 2.97)

aBeta Adjusted beta coefficient; aOR Adjusted Odds Ratio; CI Confidence interval; DBP Diastolic blood pressure; DM Diabetes mellitus; NCD: Non-communicable diseases; n_s Number of sites; n_v Number of visits; SBP Systolic blood pressure. P<0.05 highlighted in bold.

[a] Mixed effects models using random effects for patients and fixed effects for sites.

Table S4b: Impact of PIC4C implementation on random plasma glucose and diabetes control.

	RPG (mmol/L)		DM control	
All visits and sites (RPG: n_v=15,778; DM control: n_v=24,005)	Beta (95% CI)	aBeta (95% CI)[a]	OR (95% CI)	aOR (95% CI)[a]
PIC4C implementation index	**1.96 (1.49 - 2.43)**	0.46 (-0.13 - 1.05)	**0.39 (0.32 - 0.46)**	**0.59 (0.47 - 0.73)**
Sex				
Male (Reference: Female)		0.30 (0.04 - 0.56)		0.97 (0.87 - 1.09)
Age group				
45-64 (Reference 18-44 years)		**-0.84 (-1.17 - -0.51)**		**1.40 (1.21 - 1.60)**
65+		**-1.40 (-1.76 - -1.05)**		**1.84 (1.58 - 2.13)**
Comorbidity				
HTN (Reference: no HTN)		**-1.16 (-1.45 - -0.87)**		**1.49 (1.32 - 1.69)**
Years since diagnosis		**0.12 (0.09 - 0.16)**		**0.93 (0.91 - 0.94)**
Time in the programme		-0.07 (-0.17 - 0.04)		**1.12 (1.07 - 1.17)**
Co-location of NCD/HIV services				
Co-located (Reference: located separately)		-0.32 (-1.59 - 0.95)		0.90 (0.60 - 1.33)
Covid index		**1.35 (0.85 - 1.85)**		**0.81 (0.66 - 0.98)**
Proportion of new patients per month		0.00 (-1.05 - 1.05)		**1.46 (1.01 - 2.09)**
Excluding first two visits (RPG: n_v=9,202; DM control: n_v=15,402)	Beta (95% CI)	aBeta (95% CI)[a]	OR (95% CI)	aOR (95% CI)[a]
PIC4C implementation index	**1.46 (0.82 - 2.11)**	0.43 (-0.31 - 1.17)	**0.45 (0.36 - 0.58)**	**0.62 (0.47 - 0.82)**
Sex				
Male (Reference: Female)		-0.03 (-0.38 - 0.32)		1.07 (0.92 - 1.25)
Age group				
45-64 (Reference 18-44 years)		-0.32 (-0.77 - 0.14)		**1.34 (1.11 - 1.63)**
65+		**-0.71 (-1.19 - -0.23)**		**1.70 (1.38 - 2.09)**
Comorbidity				
HTN (Reference: no HTN)		-0.43 (-0.87 - 0.02)		1.10 (0.91 - 1.33)
Years since diagnosis		0.11 (0.06 - 0.15)		**0.93 (0.91 - 0.94)**
Time in the programme		**-0.34 (-0.49 - -0.19)**		**1.28 (1.20 - 1.37)**
Co-location of NCD/HIV services				
Co-located (Reference: located separately)		-0.47 (-1.89 - 0.96)		0.88 (0.56 - 1.38)
Covid index		**0.69 (0.12 - 1.27)**		1.05 (0.84 - 1.33)
Proportion of new patients per month		0.09 (-1.16 - 1.34)		1.39 (0.90 - 2.15)
Restricted to sites that implemented all seven activities (n_s=10; RPG: n_v=2,279; DM control: n_v=4,358)	Beta (95% CI)	aBeta (95% CI)[a]	Beta (95% CI)	Beta (95% CI)[a]
PIC4C implementation index	**1.84 (0.88 - 2.81)**	-0.03 (-1.24 - 1.19)	**0.23 (0.16 - 0.35)**	0.64 (0.38 - 1.09)
Sex				
Male (Reference: Female)		0.23 (-0.45 - 0.90)		1.07 (0.76 - 1.52)
Age group				
45-64 (Reference 18-44 years)		-0.00 (-0.85 - 0.85)		1.24 (0.81 - 1.89)
65+		-0.75 (-1.65 - 0.15)		**1.84 (1.17 - 2.90)**
Comorbidity				
HTN (Reference: no HTN)		-0.61 (-1.35 - 0.13)		1.13 (0.78 - 1.63)
Years since diagnosis		**0.20 (0.11 - 0.29)**		**0.84 (0.80 - 0.89)**
Time in the programme		**-0.26 (-0.52 - -0.01)**		**1.49 (1.30 - 1.70)**
Co-location of NCD/HIV services				
Co-located (Reference: located separately)		-0.91 (-2.66 - 0.83)		0.41 (0.17 - 1.01)
Covid index		**1.94 (0.88 - 3.00)**		**0.59 (0.37 - 0.94)**
Proportion of new patients per month		-0.78 (-3.02 - 1.46)		0.92 (0.34 - 2.49)
Restricted to sites that implemented all seven activities and excluding first two visits (n_s=10; RPG: n_v=1,455; DM control: n_v=3,064)	Beta (95% CI)	aBeta (95% CI)[a]	Beta (95% CI)	aBeta (95% CI)[a]
PIC4C implementation index	**2.15 (0.76 - 3.54)**	0.70 (-0.90 - 2.31)	**0.25 (0.15 - 0.42)**	**0.49 (0.26 - 0.95)**
Sex				
Male (Reference: Female)		0.14 (-0.77 - 1.05)		1.19 (0.74 - 1.91)
Age group				
45-64 (Reference 18-44 years)		0.21 (-0.89 - 1.30)		1.34 (0.77 - 2.33)
65+		-0.63 (-1.80 - 0.54)		**2.10 (1.16 - 3.80)**
Comorbidity				
HTN (Reference: no HTN)		-0.78 (-1.91 - 0.35)		1.06 (0.62 - 1.80)
Years since diagnosis		**0.21 (0.10 - 0.32)**		**0.83 (0.78 - 0.88)**
Time in the programme		**-0.50 (-0.84 - -0.16)**		**1.59 (1.33 - 1.92)**
Co-location of NCD/HIV services				
Co-located (Reference: located separately)		-0.95 (-4.10 - 2.20)		0.48 (0.11 - 2.06)
Covid index		1.08 (-0.16 - 2.32)		0.82 (0.47 - 1.41)
Proportion of new patients per month		-0.09 (-2.78 - 2.61)		1.24 (0.38 - 4.05)

aBeta Adjusted beta coefficient; aOR Adjusted Odds Ratio; BP Blood pressure; CI Confidence interval; DM Diabetes mellitus; NCD: Non-communicable diseases; n_s Number of sites; n_v Number of visits; RPG Random plasma glucose. P<0.05 highlighted in bold.

[a] Mixed effects models using random effects for patients and fixed effects for sites.

Table S5: Impact of individual PIC4C implementation components on hypertension, diabetes and viral suppression.

	BP control		DBP		DM control		RPG		Viral suppression	
	**OR (95% CI)**	**aOR (95% CI)[a]**	**Beta (95% CI)**	**Beta (95% CI)[a]**	**OR (95% CI)**	**aOR (95% CI)[b]**	**Beta (95% CI)**	**Beta (95% CI)[b]**	**OR (95% CI)**	**aOR (95% CI)[c]**
**All visits and sites**	**n_v=66,641**		**n_v=66,641**		**n_v=24,005**		**n_v=15,778**		**n_v=84,855**	
Training	0.77 (0.73-0.81)	1.07 (1.01-1.14)	0.34 (0.08-0.59)	-1.08 (-1.35--0.82)	0.70 (0.64-0.77)	0.86 (0.78-0.96)	0.75 (0.51-0.99)	0.13 (-0.14-0.39)	1.14 (1.05-1.23)	1.02 (0.93-1.11)
RFPS	0.43 (0.40-0.46)	0.66 (0.61-0.72)	5.16 (4.86-5.46)	3.50 (3.15-3.85)	0.53 (0.46-0.61)	0.67 (0.57-0.77)	1.42 (0.84-2.00)	0.72 (0.13-1.32)	1.01 (0.91-1.13)	0.85 (0.75-0.96)
Mentorship	0.64 (0.61-0.68)	0.96 (0.90-1.02)	1.48 (1.23-1.72)	-0.25 (-0.51-0.02)	0.67 (0.61-0.73)	0.84 (0.76-0.93)	0.71 (0.48-0.94)	0.13 (-0.13-0.38)	1.28 (1.19-1.39)	1.15 (1.05-1.26)
Data strengthening	0.58 (0.55-0.61)	0.93 (0.87-1.00)	2.45 (2.20-2.71)	0.12 (-0.18-0.43)	0.63 (0.58-0.69)	0.79 (0.71-0.88)	1.05 (0.81-1.30)	0.38 (0.09-0.67)	1.20 (1.11-1.30)	1.03 (0.93-1.14)
Patient support group	0.47 (0.44-0.50)	0.72 (0.66-0.78)	3.60 (3.34-3.87)	1.62 (1.26-1.97)	0.59 (0.54-0.66)	0.71 (0.62-0.82)	1.20 (0.93-1.46)	0.39 (0.01-0.76)	1.45 (1.33-1.57)	1.29 (1.13-1.48)
Equipment	0.94 (0.59-1.50)	1.29 (0.80-2.08)	-0.32 (-2.41-1.77)	-1.87 (-3.93-0.19)	1.73 (0.84-3.56)	2.24 (1.09-4.62)	-0.99 (-2.49-0.52)	-1.65 (-3.15--0.15)	--	--
Group cares	1.05 (0.94-1.18)	1.08 (0.97-1.21)	0.67 (0.17-1.17)	0.68 (0.18-1.18)	1.30 (1.08-1.57)	1.33 (1.10-1.60)	-0.64 (-1.10--0.18)	-0.64 (-1.10--0.19)	1.18 (0.98-1.42)	1.19 (0.99-1.43)
**Excluding first two visits**	**(n_v=44,901)**		**(n_v=44,901)**		**(n_v=15,402)**		**(n_v=9,202)**			
Training	0.95 (0.89-1.01)	1.11 (1.03-1.19)	-0.26 (-0.54-0.02)	-0.89 (-1.19--0.59)	0.79 (0.70-0.90)	0.92 (0.81-1.04)	0.42 (0.11-0.73)	0.04 (-0.29-0.37)		
RFPS	0.56 (0.52-0.61)	0.69 (0.63-0.76)	3.79 (3.46-4.11)	2.95 (2.57-3.33)	0.62 (0.53-0.73)	0.71 (0.60-0.85)	0.94 (0.27-1.61)	0.54 (-0.14-1.23)		
Mentorship	0.82 (0.77-0.87)	0.99 (0.92-1.06)	0.77 (0.49-1.05)	-0.01 (-0.30-0.29)	0.69 (0.61-0.78)	0.81 (0.71-0.92)	0.60 (0.30-0.90)	0.22 (-0.10-0.54)		
Data strengthening	0.76 (0.71-0.82)	1.01 (0.93-1.10)	1.33 (1.05-1.62)	-0.14 (-0.48-0.20)	0.72 (0.63-0.81)	0.84 (0.73-0.96)	0.69 (0.38-1.01)	0.27 (-0.08-0.62)		
Patient support group	0.63 (0.59-0.67)	0.79 (0.72-0.87)	2.53 (2.24-2.83)	1.36 (0.97-1.74)	0.69 (0.60-0.78)	0.75 (0.64-0.88)	0.66 (0.32-1.00)	0.11 (-0.32-0.54)		
Equipment	1.16 (0.60-2.26)	1.39 (0.72-2.71)	-0.70 (-3.59-2.19)	-1.74 (-4.61-1.13)	1.42 (0.52-3.92)	1.71 (0.63-4.67)	-0.44 (-2.39-1.51)	-0.90 (-2.84-1.05)		
Group cares	0.94 (0.80-1.10)	0.95 (0.81-1.11)	1.07 (0.39-1.75)	1.01 (0.33-1.68)	1.55 (1.18-2.04)	1.54 (1.17-2.02)	-0.36 (-1.02-0.30)	-0.33 (-0.99-0.33)		
**Restricted to sites that implemented all seven activities (n=10)**	**(n_v=13,625)**		**(n_v=13,625)**		**(n_v=4,358)**		**(n_v=2,279)**			
Training	0.63 (0.57-0.71)	1.12 (0.96-1.29)	1.39 (0.85-1.92)	-1.13 (-1.77--0.50)	0.62 (0.49-0.78)	0.99 (0.76-1.29)	0.47 (-0.08-1.01)	-0.38 (-0.99-0.23)		
RFPS	0.44 (0.38-0.49)	0.78 (0.66-0.93)	3.98 (3.41-4.55)	0.96 (0.23-1.70)	0.33 (0.25-0.44)	0.55 (0.39-0.77)	2.36 (1.49-3.24)	1.43 (0.49-2.37)		
Mentorship	0.48 (0.43-0.54)	0.92 (0.78-1.08)	3.29 (2.76-3.83)	0.03 (-0.67-0.72)	0.51 (0.40-0.64)	0.91 (0.68-1.23)	1.05 (0.50-1.59)	0.13 (-0.53-0.79)		
Data strengthening	0.51 (0.46-0.57)	0.96 (0.82-1.12)	2.49 (1.96-3.02)	-0.69 (-1.38--0.01)	0.54 (0.43-0.69)	0.91 (0.70-1.20)	0.84 (0.30-1.37)	-0.00 (-0.62-0.62)		
Patient support group	0.42 (0.38-0.48)	0.75 (0.61-0.91)	3.83 (3.30-4.36)	0.11 (-0.71-0.93)	0.41 (0.32-0.52)	0.69 (0.49-0.97)	1.29 (0.74-1.84)	0.22 (-0.56-1.00)		
Equipment*	--	--	--	--	--	--	--	--		

Group cares	1.45 (1.18-1.79)	1.53 (1.22-1.91)	-0.05 (-0.92-0.81)	0.45 (-0.48-1.38)	0.97 (0.67-1.40)	0.98 (0.68-1.43)	-0.64 (-1.31-0.03)	-0.64 (-1.32-0.04)
**Restricted to sites that implemented all seven activities (n=10) and excluding first two visits**	**(n_v=9,927)**		**(n_v=9,927)**		**(n_v=3,064)**		**(n_v=1,455)**	
Training	0.87 (0.75-1.00)	1.21 (1.02-1.44)	0.19 (-0.41-0.80)	-0.99 (-1.71--0.27)	0.71 (0.53-0.95)	0.94 (0.68-1.29)	0.27 (-0.45-1.00)	-0.29 (-1.07-0.48)
RFPS	0.53 (0.46-0.62)	0.74 (0.60-0.90)	3.24 (2.60-3.87)	1.05 (0.24-1.86)	0.36 (0.26-0.51)	0.49 (0.33-0.73)	2.44 (1.33-3.55)	1.70 (0.55-2.85)
Mentorship	0.65 (0.56-0.75)	1.03 (0.86-1.25)	2.15 (1.53-2.76)	-0.27 (-1.04-0.50)	0.55 (0.41-0.75)	0.81 (0.57-1.15)	1.15 (0.43-1.88)	0.56 (-0.26-1.37)
Data strengthening	0.66 (0.58-0.76)	1.02 (0.85-1.23)	1.45 (0.85-2.05)	-0.72 (-1.48-0.04)	0.59 (0.44-0.79)	0.81 (0.59-1.11)	0.88 (0.16-1.59)	0.34 (-0.43-1.11)
Patient support group	0.53 (0.46-0.62)	0.76 (0.60-0.95)	3.05 (2.44-3.66)	0.15 (-0.76-1.07)	0.46 (0.34-0.62)	0.63 (0.43-0.95)	1.02 (0.28-1.75)	0.15 (-0.78-1.07)
Equipment*	--	--	--	--	--	--	--	--
Group cares	1.30 (0.81-2.07)	1.27 (0.79-2.05)	-1.14 (-3.03-0.74)	-0.63 (-2.55-1.29)	1.39 (0.68-2.84)	1.26 (0.62-2.58)	-0.89 (-2.20-0.42)	-0.82 (-2.15-0.50)

BP Blood pressure; CI Confidence interval; DBP Diastolic blood pressure; DM Diabetes mellitus; FPG Fasting plasma glucose; n_v Number of visits; RPG Random plasma glucose; SBP Systolic blood pressure.

*All facilities had equipment as of January 2017.

[a] Mixed effects models using random effects for patients and fixed effects for sites adjusted for sex, age, diabetes, time in the programme, time since hypertension diagnosis, services co-location, impact of covid policies that primarily restrict people's behaviour, proportion of new patients with hypertension per month and seasonality.

[b] Mixed effects models using random effects for patients and fixed effects for sites adjusted for sex, age, hypertension, time in the programme, time since diabetes diagnosis, services co-location, impact of covid policies that primarily restrict people's behaviour, and proportion of new patients with diabetes per month.

[c] Mixed effects models using random effects for patients and fixed effects for sites adjusted for sex, age, comorbidity (diabetes, hypertension or both), time in the programme, services co-location, impact of covid policies that primarily restrict people's behaviour and proportion of new patients with hypertension or diabetes per month.

Table S6: Impact of PIC4C implementation on viral load.

	Viral load (copies/mL)		Viral suppression	
**All visits and sites (n_s=14; n_v=84,855)**	**Beta (95% CI)**	**aBeta (95% CI)[a]**	**OR**	**aOR[a]**
**PIC4C implementation index**	2060.09 (923.09-3197.08)	-1573.35 (-3163.04-16.33)	1.58 (1.36-1.83)	1.20 (0.98-1.47)
**Sex**				
Male (Reference: Female)		1825.20 (1153.26-2497.15)		0.66 (0.61-0.71)
**Age group**				
45-64 (Reference 18-44 years)		-1755.51 (-2371.32--1139.69)		1.29 (1.19-1.39)
65+		-1043.73 (-2609.18-521.72)		1.35 (1.11-1.66)
**Comorbidity**				
HTN/DM (Reference: no HTN/DM)		-1108.90 (-3804.77-1586.96)		1.32 (0.93-1.88)
DM		-1693.34 (-4624.22-1237.53)		1.24 (0.85-1.80)
HTN		-2536.67 (-16499.04-11425.71)		1.26 (0.21-7.53)
**Time in the programme**		-1151.08 (-1492.10--810.06)		0.99 (0.96-1.03)
**Covid index**		1598.43 (485.80-2711.06)		1.34 (1.14-1.57)
**Proportion of new patients**		**-2334.07 (-4081.93 - -586.21)**		2.84 (2.20-3.67)

aBeta Adjusted beta coefficient; aOR Adjusted Odds Ratio; BP Blood pressure; CI Confidence interval; DBP Diastolic blood pressure; DM Diabetes mellitus; FPG Fasting plasma glucose; n_s Number of sites; n_v Number of visits; RPG Random plasma glucose; SBP Systolic blood pressure.

[a] Mixed effects models using random effects for patients and fixed effects for sites adjusted for sex, age, comorbidity (diabetes, hypertension or both), time in the programme and impact of covid policies that primarily restrict people's behaviour.

Table S7: Impact of PIC4C implementation on systolic blood pressure and fasting plasma glucose).

	SBP (mm HG)		FPG (mmol/L)	
**Excluding first two visits (n_v=44,901)**	**Beta (95% CI)**	**Beta (95% CI)[a]**	**Beta (95% CI)**	**Beta (95% CI)[a]**
**PIC4C implementation index**	**5.02 (4.08 - 5.95)**	**1.39 (0.28 - 2.49)**	**1.42 (0.93 - 1.91)**	**0.72 (0.12 - 1.32)**
**Sex**				
Male (Reference: Female)		**1.48 (0.66 - 2.29)**		-0.28 (-0.66 - 0.09)
**Age group**				
45-64 (Reference 18-44 years)		0.68 (-0.30 - 1.65)		**-0.83 (-1.27 - -0.38)**
65+		**3.88 (2.85 - 4.92)**		**-1.22 (-1.71 - -0.74)**
**Comorbidity**				
DM (Reference: no DM)		-6.94 (-16.41 - 2.54)		-0.37 (-0.79 - 0.06)
HTN (Reference: no HTN)				
**Years since diagnosis**		**0.16 (0.08 - 0.25)**		**0.10 (0.06 - 0.14)**
**Time in the programme**		**-0.99 (-1.30 - -0.68)**		**-0.47 (-0.63 - -0.31)**
**Co-location of NCD/HIV services**				
Co-located (Reference: located separately)		-1.41 (-3.01 - 0.18)		-0.19 (-1.02 - 0.64)
**Covid index**		**4.14 (3.26 - 5.02)**		0.21 (-0.32 - 0.74)
**Proportion of new patients per month**		1.11 (-0.32 - 2.53)		-0.65 (-1.54 - 0.24)
**Restricted to sites that implemented all seven activities (n_s=10; n_v=13,625)**	**Beta (95% CI)**	**aBeta (95% CI)[a]**	**Beta (95% CI)**	**aBeta (95% CI)[a]**
**PIC4C implementation index**	**8.97 (7.46 - 10.48)**	0.95 (-1.17 - 3.07)	**2.12 (1.40 - 2.83)**	0.42 (-0.59 - 1.44)
**Sex**				
Male (Reference: Female)		0.94 (-0.60 - 2.49)		-0.32 (-0.96 - 0.33)
**Age group**				
45-64 (Reference 18-44 years)		-0.88 (-2.74 - 0.99)		**-0.95 (-1.74 - -0.16)**
65+		**2.51 (0.56 - 4.46)**		**-1.88 (-2.72 - -1.04)**
**Comorbidity**				
DM (Reference: no DM)		1.56 (-6.69 - 9.81)		-0.35 (-1.03 - 0.32)
HTN (Reference: no HTN)				
**Years since diagnosis**		0.06 (-0.15 - 0.28)		**0.17 (0.09 - 0.25)**
**Time in the programme**		**-2.71 (-3.23 - -2.19)**		**-0.57 (-0.82 - -0.31)**
**Co-location of NCD/HIV services**				
Co-located (Reference: located separately)		-0.15 (-3.46 - 3.16)		2.55 (-0.50 - 5.59)
**Covid index**		**5.84 (4.04 - 7.65)**		0.90 (-0.05 - 1.85)
**Proportion of new patients per month**		-1.10 (-4.59 - 2.39)		**-2.17 (-4.11 - -0.23)**

aBeta Adjusted beta coefficient; CI Confidence interval; DBP Diastolic blood pressure; DM Diabetes mellitus; NCD: Non-communicable diseases; n_s Number of sites; n_v Number of visits; SBP Systolic blood pressure. P<0.05 highlighted in bold.

[a] Mixed effects models using random effects for patients and fixed effects for sites.

Table S8: Impact of individual PIC4C implementation on systolic blood pressure and fasting plasma glucose.

	SBP		FPG	
**Excluding first two visits**	**(n_v=44,901)**		**(n_v=6,217)**	
Training	**0.49 (0.01-0.97)**	-0.46 (-0.98-0.05)	**0.39 (0.11-0.66)**	0.11 (-0.18-0.40)
RFPS	**3.79 (3.24-4.35)**	**2.11 (1.46-2.75)**	**0.87 (0.58-1.16)**	**0.56 (0.20-0.92)**
Mentorship	**1.71 (1.23-2.19)**	**0.52 (0.01-1.03)**	**0.66 (0.38-0.94)**	0.26 (-0.05-0.57)
Data strengthening	**1.97 (1.49-2.46)**	-0.24 (-0.82-0.34)	**0.59 (0.32-0.86)**	0.20 (-0.10-0.50)
Patient support group	**3.42 (2.92-3.93)**	**1.16 (0.50-1.83)**	**0.88 (0.60-1.17)**	**0.62 (0.25-0.98)**
Equipment	0.45 (-4.48-5.38)	-0.74 (-5.65-4.17)	5.35 (-1.19-11.89)	5.69 (-0.74-12.13)
Group cares	0.89 (-0.27-2.05)	1.04 (-0.11-2.20)	**-0.70 (-1.38 - -0.02)**	-0.61 (-1.29-0.07)
**Restricted to sites that implemented all seven activities (n=10)**	**(n_v=13,625)**		**(n_v=2,082)**	
Training	**3.00 (2.11-3.89)**	**-1.12 (-2.19--0.04)**	**0.81 (0.38-1.24)**	-0.06 (-0.56-0.43)
RFPS	**5.89 (4.93-6.85)**	**1.88 (0.64-3.13)**	**1.38 (0.93-1.83)**	0.53 (-0.14-1.19)
Mentorship	**5.10 (4.21-6.00)**	0.64 (-0.54-1.82)	**1.12 (0.66-1.58)**	0.07 (-0.52-0.66)
Data strengthening	**4.81 (3.93-5.69)**	0.78 (-0.39-1.95)	**1.14 (0.71-1.57)**	0.25 (-0.28-0.77)
Patient support group	**6.15 (5.26-7.04)**	**2.51 (1.10-3.91)**	**1.39 (0.95-1.84)**	0.55 (-0.11-1.22)
Equipment*	--	--	--	--
Group cares	**-2.27 (-3.74--0.80)**	**-2.67 (-4.25--1.09)**	-1.18 (-2.60-0.25)	-0.81 (-2.21-0.59)

BP Blood pressure; CI Confidence interval; DBP Diastolic blood pressure; DM Diabetes mellitus; FPG Fasting plasma glucose; n_v Number of visits; RPG Random plasma glucose; SBP Systolic blood pressure.

P<0.05 highlighted in bold.

*All facilities had equipment as of January 2017.

[a] Mixed effects models using random effects for patients and fixed effects for sites adjusted for sex, age, diabetes, time in the programme, time since hypertension diagnosis, services co-location, impact of covid policies that primarily restrict people's behaviour, proportion of new patients with hypertension per month and seasonality.

[b] Mixed effects models using random effects for patients and fixed effects for sites adjusted for sex, age, hypertension, time in the programme, time since diabetes diagnosis, services co-location, impact of covid policies that primarily restrict people's behaviour, and proportion of new patients with diabetes per month.

[c] Mixed effects models using random effects for patients and fixed effects for sites adjusted for sex, age, comorbidity (diabetes, hypertension or both), time in the programme, services co-location, impact of covid policies that primarily restrict people's behaviour and proportion of new patients with hypertension or diabetes per month

Table S9: Impact of PIC4C implementation index on hypertension, diabetes and viral suppression using unequal weights.

	Primary outcomes				Secondary outcomes											
	SBP		FPG		BP control		DBP		DM control		RPG		Viral suppression		Viral load (copies/mL)	
	Beta (95% CI)	Beta (95% CI)[a]	Beta (95% CI)	Beta (95% CI)[b]	OR (95% CI)	aOR (95% CI)[a]	Beta (95% CI)	Beta (95% CI)[a]	OR (95% CI)	aOR (95% CI)[b]	Beta (95% CI)	Beta (95% CI)[b]	OR (95% CI)	aOR (95% CI)[c]	Beta (95% CI)	Beta (95% CI)[c]
**All visits and sites**	**n_v=66,641**				**n_v=8,268**				**n_v=24,005**		**n_v=15,778**		**n_v=84,855**		**n_v=84,855**	
Implementation index (Unequal weights)*	10.60 (9.78-11.43)	2.62 (1.59-3.66)	1.52 (1.11-1.94)	0.65 (0.10-1.20)	0.28 (0.25-0.31)	0.67 (0.58-0.77)	6.58 (6.09-7.06)	2.54 (1.93-3.14)	0.36 (0.30-0.44)	0.55 (0.44-0.70)	2.19 (1.67-2.72)	0.49 (-0.18-1.16)	1.65 (1.41-1.92)	1.22 (0.97-1.52)	2318 (1133-3503)	-1443 (-3173-286)
**Excluding first two visits**	**(n_v=44,901)**				**(n_v=6,217)**				**(n_v=15,402)**		**(n_v=9,202)**					
Implementation index (Unequal weights)*	5.82 (4.87-6.77)	2.19 (1.03-3.35)	1.45 (0.96-1.93)	0.80 (0.19-1.41)	0.46 (0.40-0.52)	0.73 (0.62-0.86)	4.48 (3.92-5.03)	2.39 (1.71-3.06)	0.45 (0.35-0.57)	0.60 (0.44-0.80)	1.61 (0.90-2.32)	0.45 (-0.38-1.29)				
**Restricted to sites that implemented all seven activities (n=10)**	**(n_v=13,625)**				**(n_v=2,082)**				**(n_v=4,358)**		**(n_v=2,279)**					
Implementation index (Unequal weights)*	9.34 (7.82-10.86)	1.36 (-0.81-3.53)	2.16 (1.45-2.88)	0.54 (-0.51-1.58)	0.28 (0.23-0.34)	0.88 (0.66-1.19)	6.13 (5.21-7.04)	0.12 (-1.16-1.40)	0.21 (0.14-0.31)	0.55 (0.32-0.96)	2.02 (1.01-3.04)	0.08 (-1.21-1.36)				
**Restricted to sites that implemented all seven activities (n=10) and excluding first two visits**	**(n_v=9,927)**				**(n_v=1,610)**				**(n_v=3,064)**		**(n_v=1,455)**					
Implementation index (Unequal weights)*	6.43 (4.63-8.23)	2.80 (0.29-5.31)	2.03 (1.13-2.92)	0.75 (-0.44-1.94)	0.40 (0.31-0.51)	0.81 (0.57-1.16)	4.44 (3.37-5.51)	-0.09 (-1.57-1.40)	0.22 (0.13-0.38)	0.42 (0.21-0.83)	2.45 (0.97-3.93)	0.86 (-0.87-2.59)				

BP Blood pressure; CI Confidence interval; DBP Diastolic blood pressure; DM Diabetes mellitus; FPG Fasting plasma glucose; n_v Number of visits; RPG Random plasma glucose; SBP Systolic blood pressure.

*20% weight on RFPS, Group cares and patient support group. 10% weight on training, equipment, mentorship and data strengthening.

[a] Mixed effects models using random effects for patients and fixed effects for sites adjusted for sex, age, diabetes, time in the programme, time since hypertension diagnosis, services co-location, impact of covid policies that primarily restrict people's behaviour, proportion of new patients with hypertension per month and seasonality.

[b] Mixed effects models using random effects for patients and fixed effects for sites adjusted for sex, age, hypertension, time in the programme, time since diabetes diagnosis, services co-location, impact of covid policies that primarily restrict people's behaviour, and proportion of new patients with diabetes per month.

[c] Mixed effects models using random effects for patients and fixed effects for sites adjusted for sex, age, comorbidity (diabetes, hypertension or both), time in the programme, services co-location, impact of covid policies that primarily restrict people's behaviour and proportion of new patients with hypertension or diabetes per month.
