# Supplementary figures and images for: Evaluating the implementation of the Primary Health Integrated Care Project for Chronic Conditions: a cohort study from Kenya
Source: BMJ Public Health. 2024 Mar 25;2(1):e000146. doi: 10.1136/bmjph-2023-000146 (PMC7616119; doi:10.1136/bmjph-2023-000146)

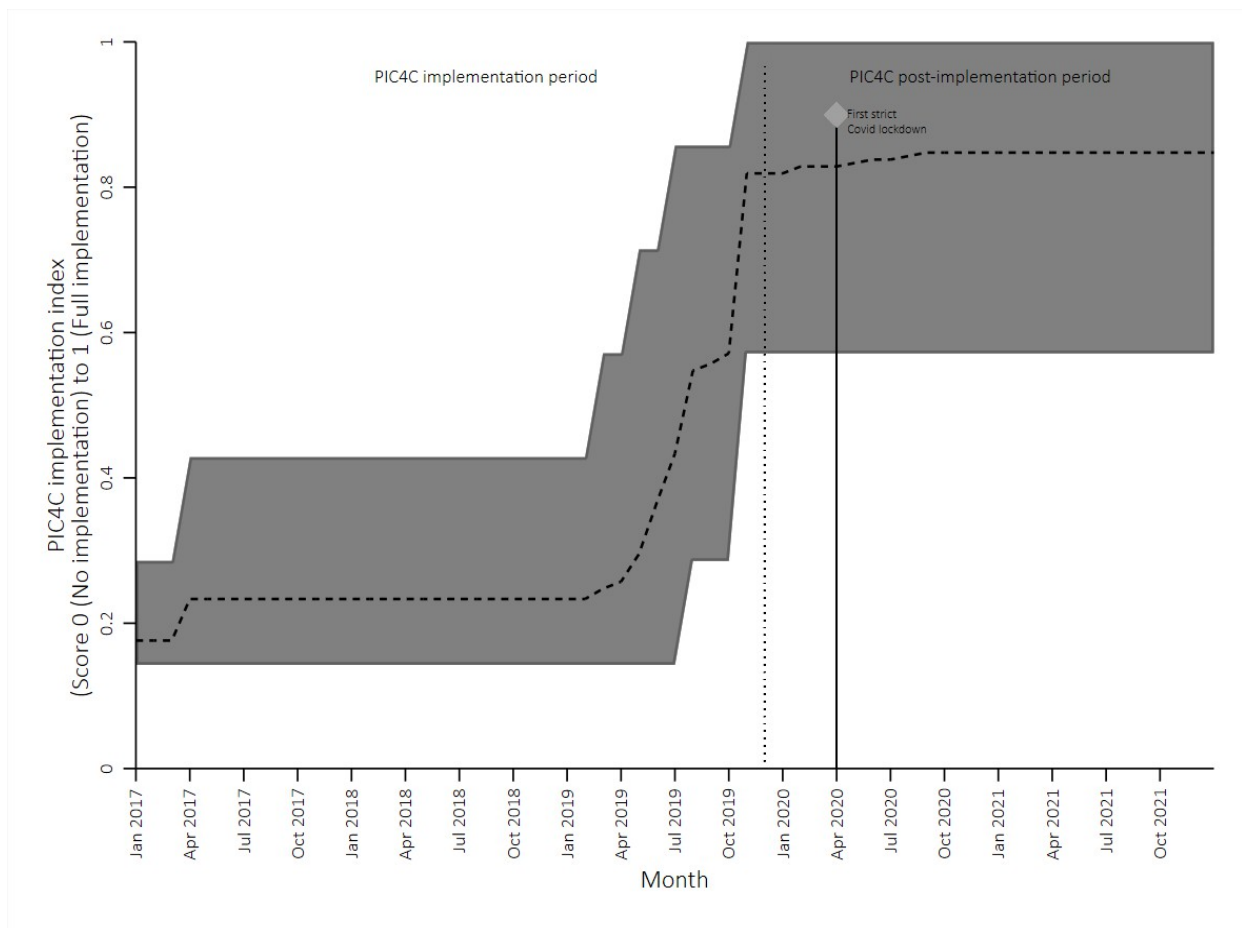

Figure S1. PIC4C implementation index

Supplement: online supplemental file 2 [file bmjph-2-1-s002.pdf]

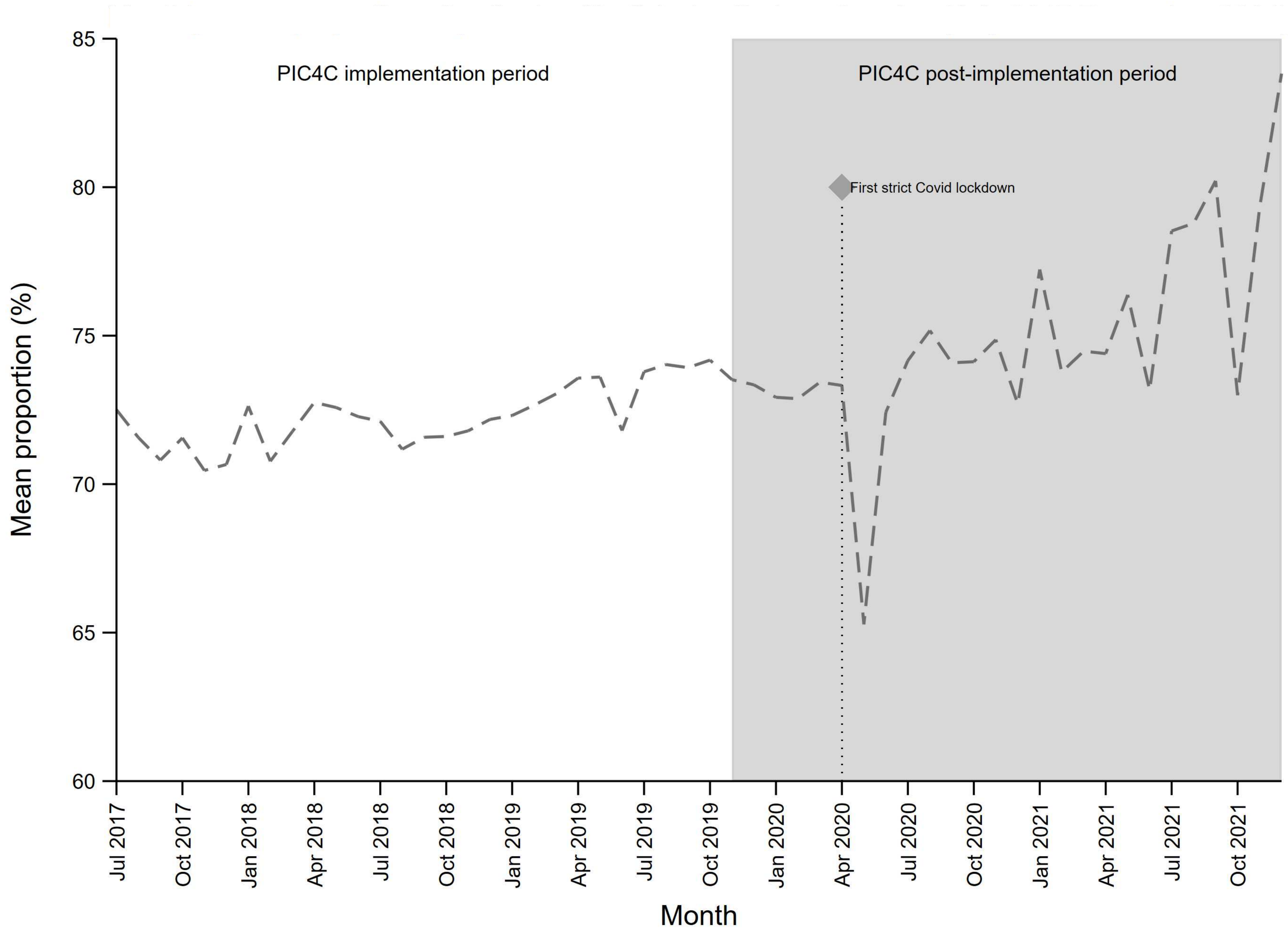

Figure S4. Monthly mean proportion of female patients with diabetes (July 2017-December 2021)

Supplement: online supplemental file 5 [file bmjph-2-1-s005.pdf]

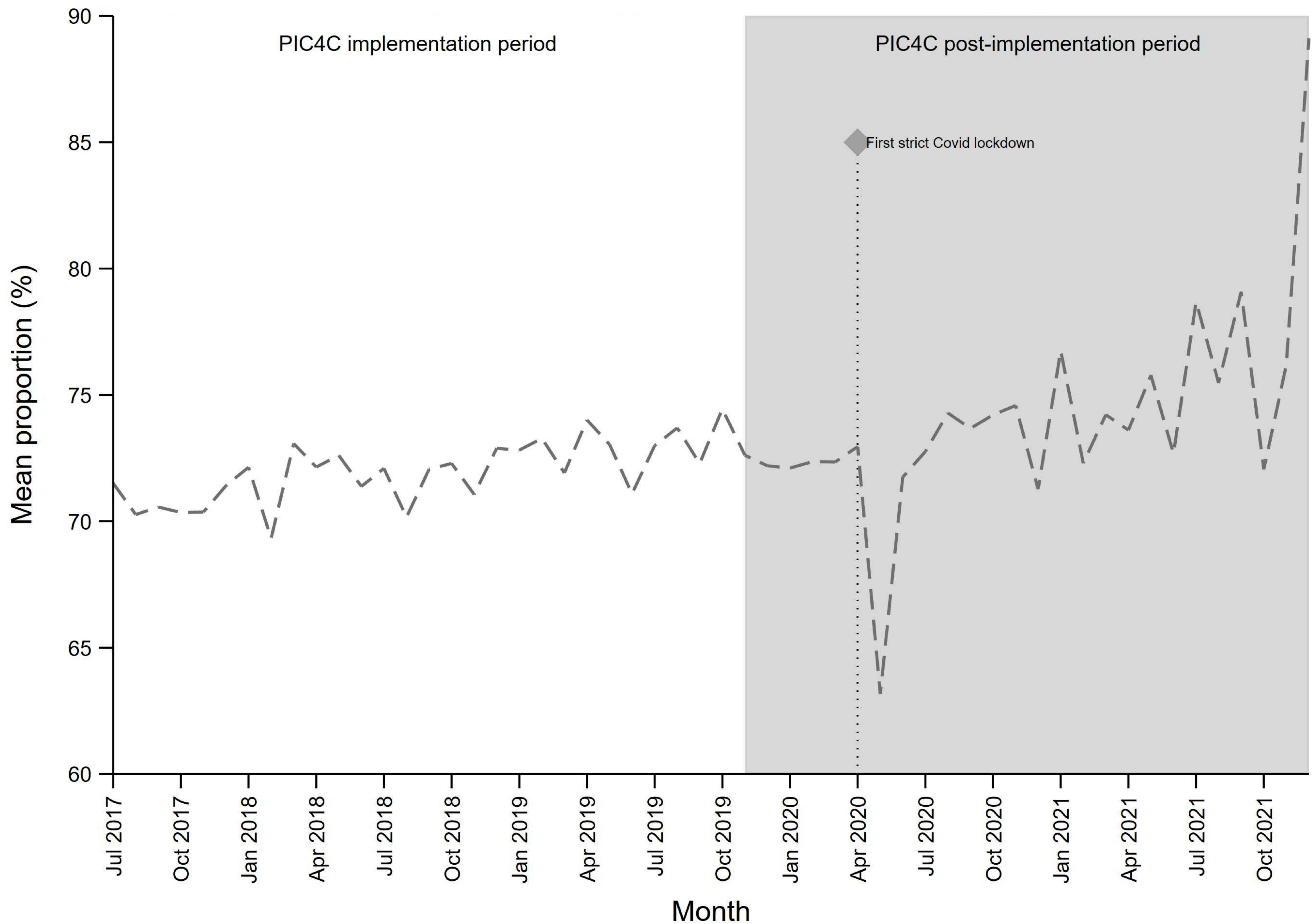

Figure S5: Monthly mean proportion of female patients with hypertension (July 2017-December 2021)

Supplement: online supplemental file 6 [file bmjph-2-1-s006.pdf]

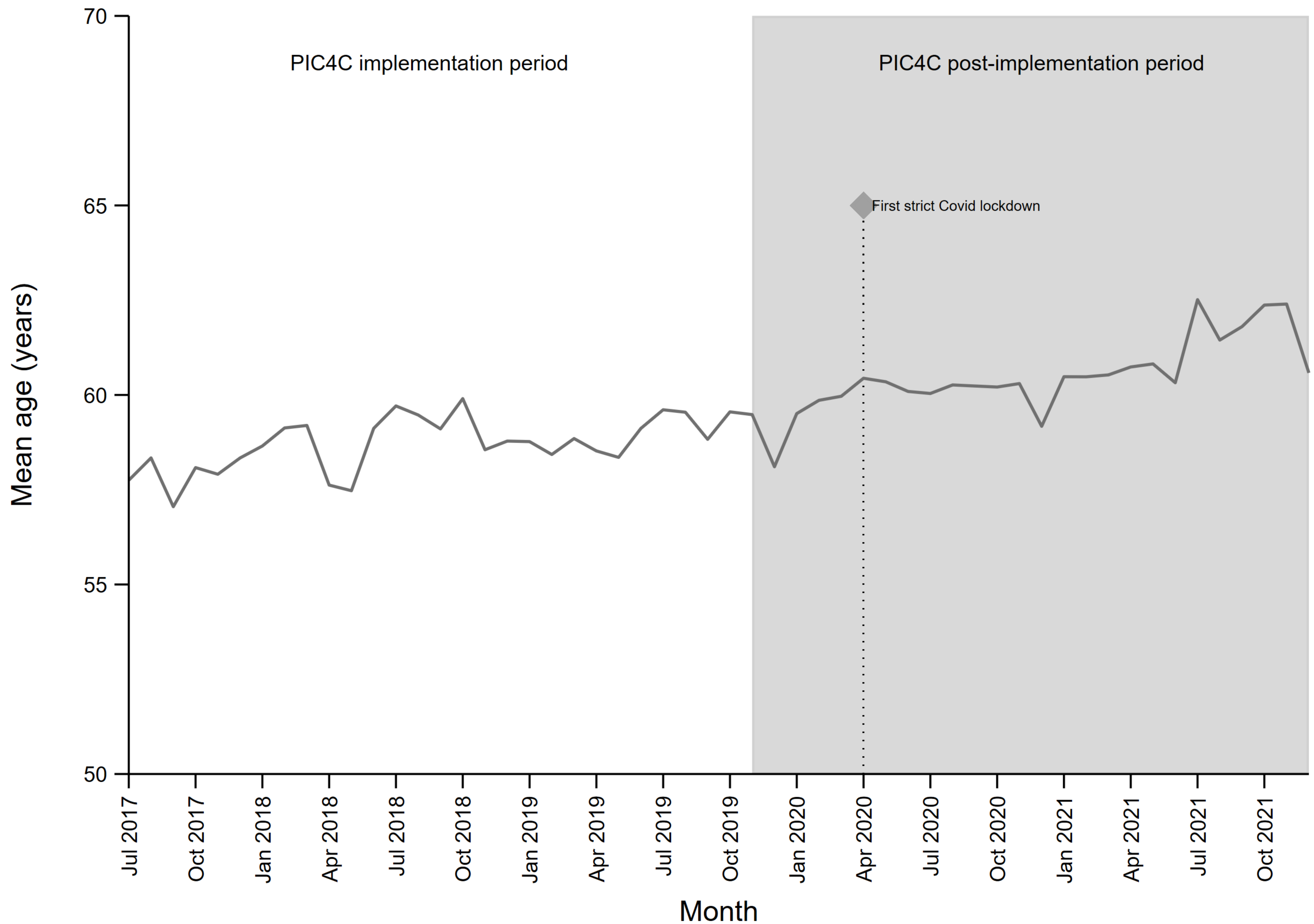

Figure S6: Monthly mean age of patients with diabetes (July 2017-December 2021)

Supplement: online supplemental file 7 [file bmjph-2-1-s007.pdf]

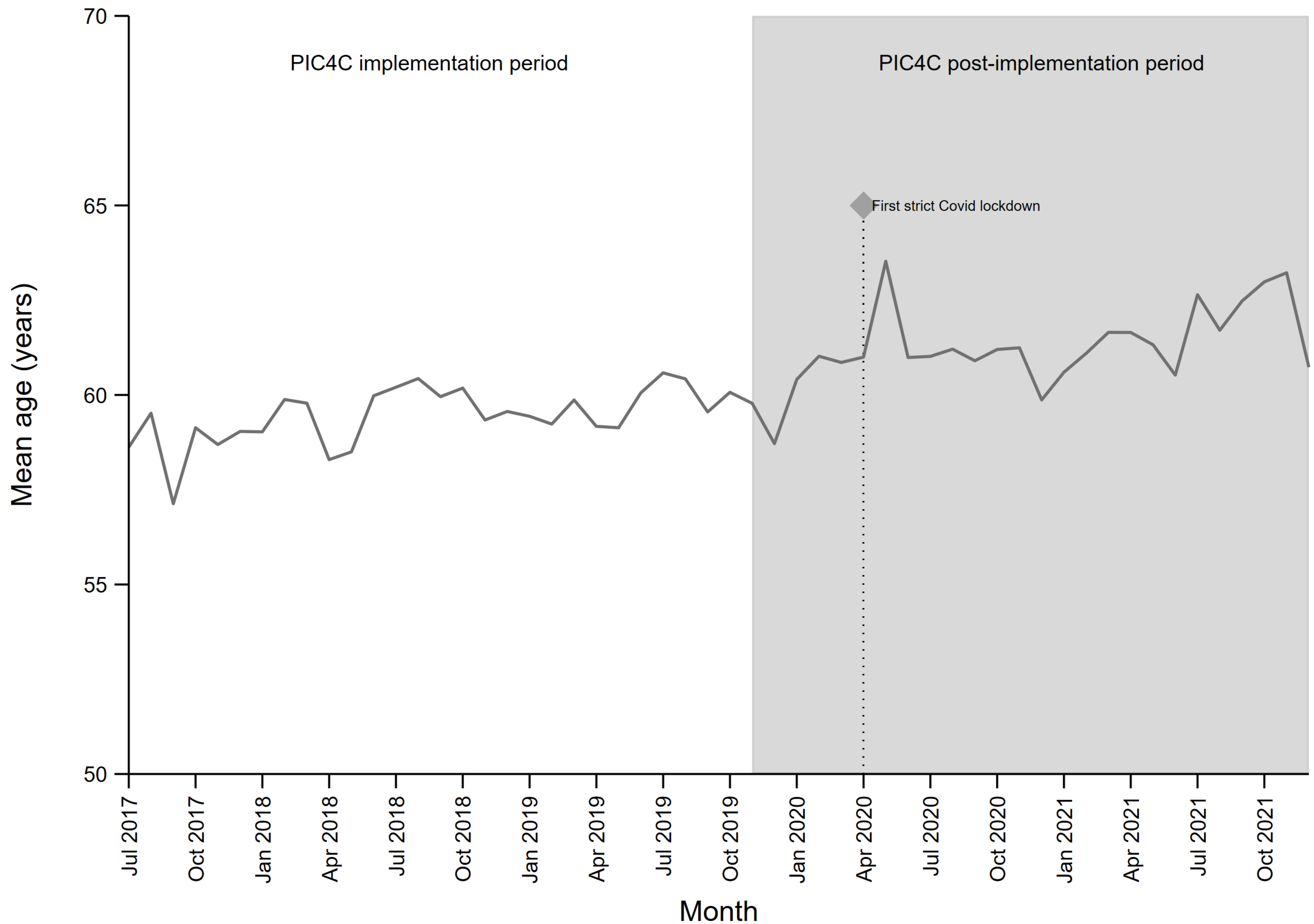

Figure S7. Monthly mean age of patients with hypertension (July 2017-December 2021)

Supplement: online supplemental file 8 [file bmjph-2-1-s008.pdf]

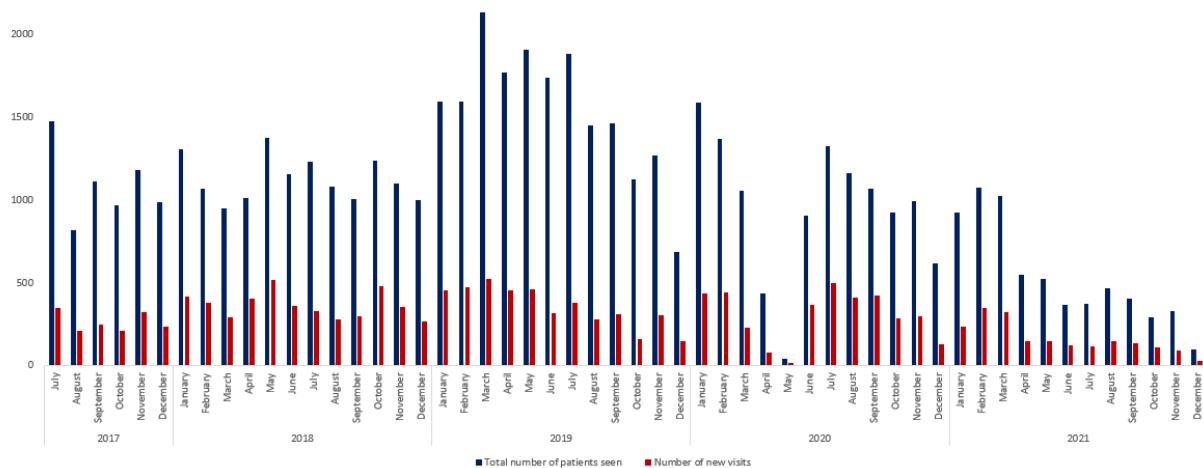

**Figure S9:** Number of all and new patients with diabetes seen across all 30 PIC4C facilities per month

Supplement: online supplemental file 10 [file bmjph-2-1-s010.pdf]
