# Supplementary material for: Evaluating the implementation of the Primary Health Integrated Care Project for Chronic Conditions: a cohort study from Kenya
Source: BMJ Public Health. 2024 Mar 25;2(1):e000146. doi: 10.1136/bmjph-2023-000146 (PMC7616119; doi:10.1136/bmjph-2023-000146)
Supplement: online supplemental file 3 [file bmjph-2-1-s003.pdf]

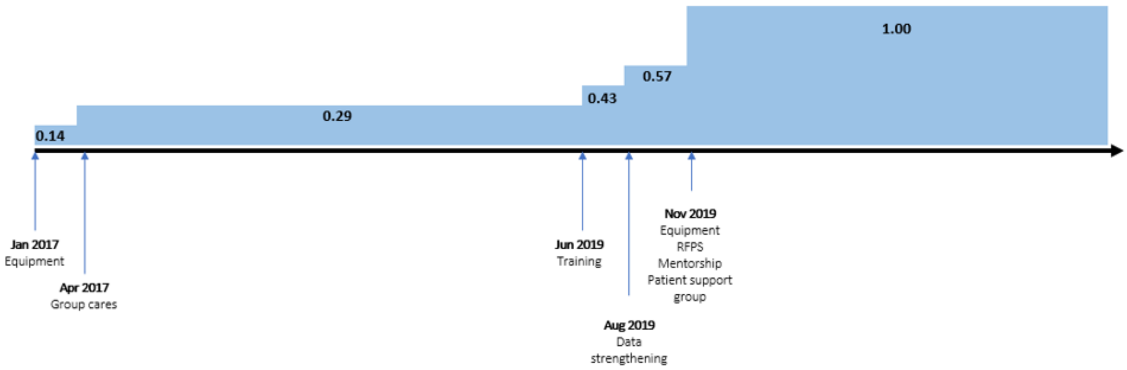

- Developed based on seven implementation activities: Training, Equipment, RFPS, Mentorship, Data Strengthening, Group Cares and Patient Support group
- Equal weights (0.143 for each activity)
- Score from 0 (no activities) to 1 (all activities)

Figure S2: Example of how the index with equal weights is calculated and captured over the course of the study facility in a facility
