# Supplementary material for: Evaluating the implementation of the Primary Health Integrated Care Project for Chronic Conditions: a cohort study from Kenya
Source: BMJ Public Health. 2024 Mar 25;2(1):e000146. doi: 10.1136/bmjph-2023-000146 (PMC7616119; doi:10.1136/bmjph-2023-000146)
Supplement: online supplemental file 4 [file bmjph-2-1-s004.pdf]

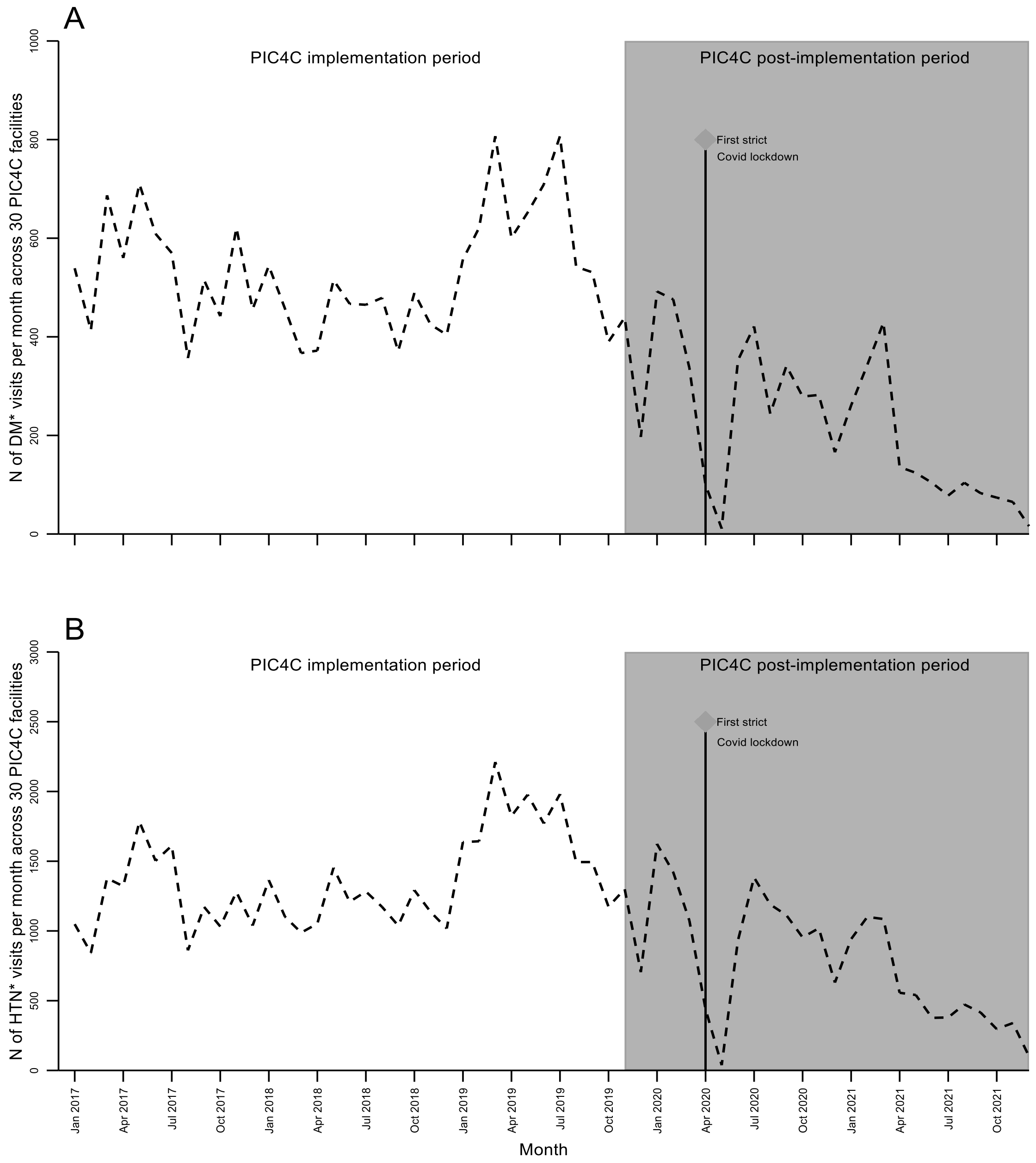

DM: Diabetes mellitus; HTN: Hypertension.

*Recorded visits with valid systolic and diastolic pressure measurement or valid fasting or random plasma glucose measurements.

Figure S3. Change in the total number of visits across 30 PIC4C facilities among patients with (a) hypertension and (b) diabetes between January 2017 and December 2021
