# Supplementary material for: Evaluating the implementation of the Primary Health Integrated Care Project for Chronic Conditions: a cohort study from Kenya
Source: BMJ Public Health. 2024 Mar 25;2(1):e000146. doi: 10.1136/bmjph-2023-000146 (PMC7616119; doi:10.1136/bmjph-2023-000146)
Supplement: online supplemental file 9 [file bmjph-2-1-s009.pdf]

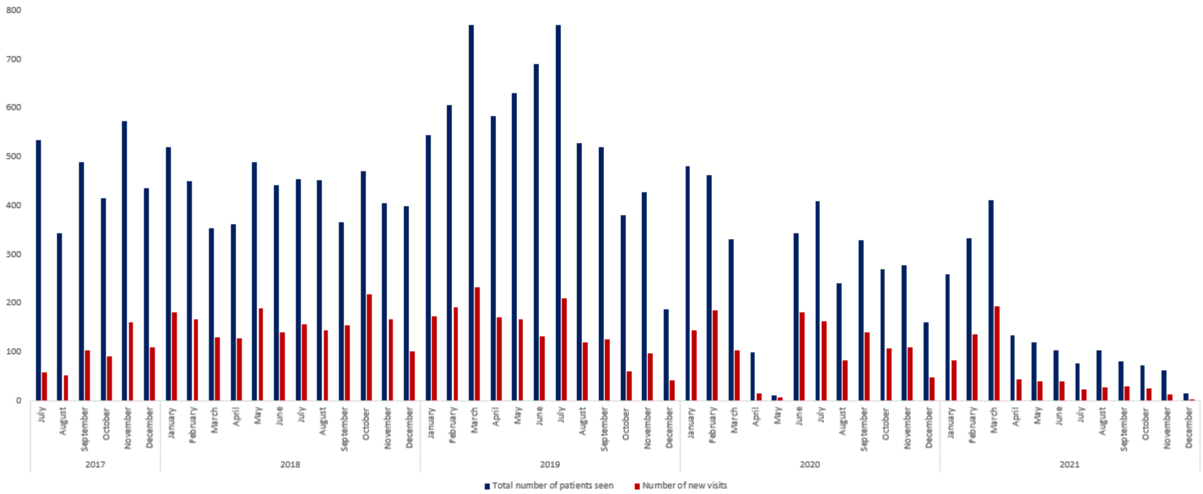

Figure S8: Number of all and new patients with hypertension seen across all 30 PIC4C facilities per month
