# Supplementary material for: Evaluating the implementation of the Primary Health Integrated Care Project for Chronic Conditions: a cohort study from Kenya
Source: BMJ Public Health. Author manuscript; Available in PMC 2024 Jun 27. (PMC7616119; doi:10.1136/bmjph-2023-000146)
Supplement: Supplementary file 2 [file EMS196781-supplement-Figure_S9.pdf]

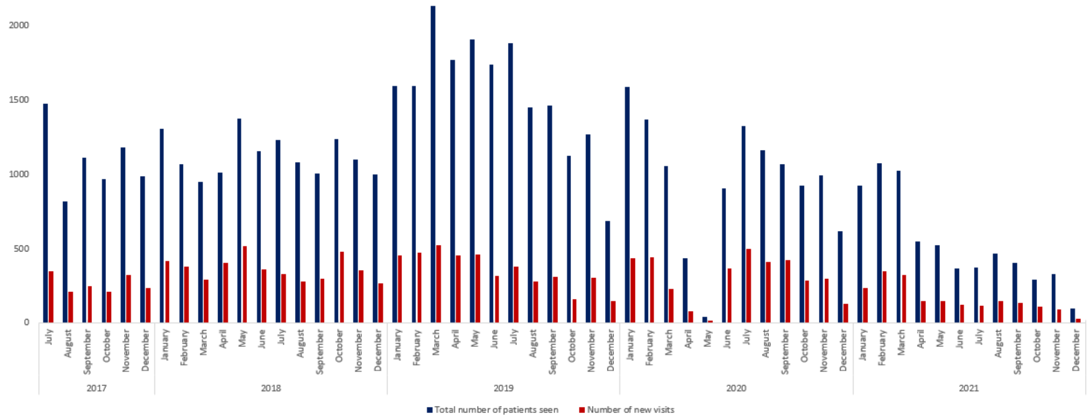

Figure S9: Number of all and new patients with diabetes seen across all 30 PIC4C facilities per month
