# Supplementary material for: Evaluating the implementation of the Primary Health Integrated Care Project for Chronic Conditions: a cohort study from Kenya
Source: BMJ Public Health. Author manuscript; Available in PMC 2024 Jun 27. (PMC7616119; doi:10.1136/bmjph-2023-000146)
Supplement: Supplementary file 3 [file EMS196781-supplement-Supplementary_tables.pdf]

**Table S1:** PIC4C Implementation activities

| Activity | Strategy | Personnel | Inputs | Outputs | Outcome |
|---|---|---|---|---|---|
| Screening | Community mobile screening booths | Community health promoters (CHP) and CHV | -CHV/CHP trainings<br>-Screening equipment<br>-Mobile phones for data collection<br>-Data bundles<br>-Manual national registers<br>-Transport facilitation supervisors/mentor time effort | Number of people educated, screened, and referred<br>Number of those referred who linked to care<br>CHV/CHP retention rates<br>Evaluation data | -Change in community knowledge<br>-Change in screening rates and Linkage rates<br>-Data for informed decision making/planning |
| | Facility triage site screening | CHP<br>Nurses<br>HRIOs | Training<br>Equipment<br>Mobile tablets for data collection<br>Manual Registers<br>Screening trainings | Number screened<br>Number referred to clinic after diagnosis | Change in triage screening rates<br>Change in practice |
| HCWs training | Classroom structured training and online CPDs | Nurses<br>Clinical officers<br>Pharmaceutical techs<br>Health record Information Officers (HRIOs)<br>Medical officers<br>Nutritionists | Trainers<br>Training curriculum<br>Training facilitation<br>Information and education materials<br>Internet bundles for CPDs | Number of trainings and online CPDs<br>Number of health workers trained<br>Topics covered in trainings | Change in knowledge measured by pre and post test<br>Change in attitude assessed through baseline and End-line Likert scale test |
| | On job mentorship | Nurses<br>Clinical officers<br>Pharmaceutical techs<br>Health record Information Officers (HRIOs)<br>Medical officers<br>Nutritionists | County based clinical mentors time effort<br>Mentorship transport<br>Mentors training | Mentorship reports<br>Support supervision reports | Improved diagnostic and management decision making skills measured through process evaluation |
| Supply chain | Revolving Fund Pharmacies | Pharmacists<br>Pharmaceutical technologists<br>Cashiers<br>Dispensary nurses | Space renovation and set up<br>MOU signing meetings with County officials and community reps<br>Seed stock of drugs<br>Computer/tablets for data capture<br>Transport and facilitation for supervision and mentorship | Number of prescription served<br>Financial reports<br>Stock rates | Increased availability of medication<br><br>Reduced cost of accessing medication<br>As per final qualitative evaluation |

| | | | | | |
|---|---|---|---|---|---|
| Patient management and follow up | Treatment in integrated primary care NCD clinics ((separate from the HIV clinics) at level 4 and 3 facilities by clinical officers, visiting mentor medical officers or consultants<br><br>Treatment in general clinics at level 2 by dispensary nurse or visiting clinical officer mentor | Clinical officers<br>Medical officers<br>Consultant physicians<br>Nurses<br>Health records information officers<br>Nutritionists<br>CHPs for triage and education<br>Lab techs<br><br>Nurses<br>CHPs/CHVs<br>Occassionally Visiting mentor<br>Clinical officers | Equipment such as Omron automatic BP machines, weight meters, height meters, glucose meters, HbA1c machine, Stethoscopes,<br><br>Data tools: Patient files, MoH Registers, Booking cards, and referral cards, Mobile tablets for electronic data capture<br><br>Healthcare workers training and mentorship | Number of new and returning patients treated<br><br>Number of patients given health education<br><br>Number of patients referred upwards for review<br><br>Number of patients referred to support groups | Blood pressure control rates<br><br>Blood glucose control rates<br><br>Adherence rates ( self reported)<br><br>Retention rates |
| Leadership and governance strengthening | Engagement for development and signing of MoU<br><br>Data Review meetings<br><br>Facility board management teams leadership trainings<br><br>Result dissemination meetings | Health facility leadership<br>Sub-county leadership<br>County health leadership<br>County health committee leadership<br>National NCD leadership<br>National health insurance fund local leadership<br>Medical Professional bodies leadership | Meeting facilitation<br><br>Training facilitation | Minutes of meetings<br><br>Memorundums of understanding<br><br>Training attendance lists | Increased adoption of project strategies into mainstream practice |
| Patient self management support | Patient support groups | Patient peer leaders<br>Patients<br>Community health workers and promoters<br>Nurses<br>Clinical officers<br>Research assistants | Training<br>Equiping with self monitoring equipment eg Bp machines, glucose meters<br>Support group registers | Number of support groups<br>Number of meetings<br>Topics covered in health education<br>Data records | Increased adherence<br>Increased retention<br>Improved treatment outcomes |

**Table S2:** Total number of visits across the study period in 28 PIC4C facilities with data on viral load.

| PIC4C site | N of visits | N of patients* |
|---|---|---|
| Aboloi Dispensary | 142 | 38 |
| Akichelesit Dispensary | 117 | 17 |
| Angurai Health Centre | 2,670 | 687 |
| Bumala A Health Centre | 7,330 | 1738 |
| Bumala B Health Centre | 3,207 | 845 |
| Changara Dispensary | 503 | 192 |
| Kamolo Dispensary | 112 | 49 |
| Khunyangu Sub County Hospital | 1,279 | 302 |
| Kitale County Hospital | 38,895 | 9925 |
| Malaba Dispensary | 1,702 | 625 |
| Moding Health Centre | 24 | 8 |
| Mukhobola Health Centre | 4,919 | 1359 |
| Port victoria Sub County Hospital | 12,820 | 3195 |
| Saboti Sub County Hospital | 5,750 | 1483 |
| Budalangi dispensary | 6 | 2 |
| Busagwa dispensary | 20 | 4 |
| Endebes health centre | 5 | 1 |
| Ikonzo dispensary | 28 | 6 |
| Kapsara sub-district hospital | 16 | 2 |
| Kwanza health centre | 2 | 2 |
| Matayos health centre | 5,409 | 1378 |
| Matunda health centre | 1 | 1 |
| Nasewa health centre | 21 | 4 |
| Rukala dispensary | 6 | 1 |
| Sikarira dispensary | 11 | 3 |
| Sirimba mission hospital | 12 | 2 |
| Sisenye dispensary | 10 | 2 |
| Sister freda medical centre | 3 | 1 |
| **Total** | **85,020** | **21,872** |

*636 patients had visits in two or more different facilities.

**Table S3**: Weights given for each activity for the index with the equal and unequal weights.

| Implementation activity | Equal weight | Unequal weight |
|---|---|---|
| Training | 0.143 | 0.1 |
| Equipment | 0.143 | 0.1 |
| RFPS | 0.143 | 0.2 |
| Mentorship | 0.143 | 0.1 |
| Data Strengthening | 0.143 | 0.1 |
| Group Cares | 0.143 | 0.2 |
| Patient Support group | 0.143 | 0.2 |
| Total (Implementation of all activities) | 1 | 1 |

**Table S4a:** Impact of PIC4C implementation on diastolic blood pressure and blood pressure control.

| | DBP (mm HG) | | BP control | |
|---|---|---|---|---|
| All visits and sites ($n_v$=66,641) | Beta (95% CI) | Beta (95% CI)[a] | OR (95% CI) | aOR (95% CI)[a] |
| PIC4C implementation index | **5.48 (5.01 - 5.96)** | **1.20 (0.62 - 1.77)** | **0.31 (0.28 - 0.35)** | **0.77 (0.68 - 0.88)** |
| Sex | | | | |
| Male (Reference: Female) | | -0.03 (-0.43 - 0.36) | | **0.84 (0.78 - 0.90)** |
| Age group | | | | |
| 45-64 (Reference 18-44 years) | | **-1.08 (-1.55 - -0.60)** | | 0.98 (0.90 - 1.07) |
| 65+ | | **-4.08 (-4.59 - -3.58)** | | **0.85 (0.78 - 0.94)** |
| Comorbidity | | | | |
| DM (Reference: no DM) | | **-2.01 (-3.76 - -0.27)** | | **1.74 (1.15 - 2.64)** |
| Years since diagnosis | | **0.05 (0.00 - 0.10)** | | 0.99 (0.98 - 1.00) |
| Time in the programme | | **-1.82 (-1.96 - -1.68)** | | **1.33 (1.29 - 1.36)** |
| Co-location of NCD/HIV services | | | | |
| Co-located (Reference: located separately) | | -0.77 (-1.65 - 0.12) | | 1.13 (0.93 - 1.36) |
| Covid index | | **3.75 (3.27 - 4.23)** | | **0.51 (0.45 - 0.57)** |
| Proportion of new patients per month | | **-2.21 (-2.97 - -1.45)** | | 1.10 (0.92 - 1.31) |
| Excluding first two visits ($n_v$=44,901) | Beta (95% CI) | Beta (95% CI)[a] | OR (95% CI) | aOR (95% CI)[a] |
| PIC4C implementation index | **3.50 (2.95 - 4.05)** | **1.22 (0.57 - 1.86)** | **0.52 (0.46 - 0.59)** | **0.84 (0.72 - 0.98)** |
| Sex | | | | |
| Male (Reference: Female) | | **-0.70 (-1.19 - -0.22)** | | **0.86 (0.78 - 0.94)** |
| Age group | | | | |
| 45-64 (Reference 18-44 years) | | -0.31 (-0.88 - 0.27) | | 0.96 (0.86 - 1.08) |
| 65+ | | **-2.82 (-3.43 - -2.21)** | | **0.84 (0.74 - 0.94)** |
| Comorbidity | | | | |
| DM (Reference: no DM) | | 0.59 (-4.99 - 6.17) | | 1.70 (0.49 - 5.92) |
| Years since diagnosis | | **0.08 (0.03 - 0.13)** | | **0.98 (0.97 - 0.99)** |
| Time in the programme | | **-0.62 (-0.80 - -0.43)** | | **1.12 (1.08 - 1.17)** |
| Co-location of NCD/HIV services | | | | |
| Co-located (Reference: located separately) | | **-1.28 (-2.21 - -0.34)** | | 1.05 (0.85 - 1.30) |
| Covid index | | **2.98 (2.47 - 3.49)** | | **0.60 (0.53 - 0.68)** |
| Proportion of new patients per month | | **-1.66 (-2.49 - -0.83)** | | 1.16 (0.95 - 1.43) |
| Restricted to sites that implemented all seven activities ($n_s$=10; $n_v$=13,625) | Beta (95% CI) | aBeta (95% CI)[a] | Beta (95% CI) | Beta (95% CI)[a] |
| PIC4C implementation index | **5.72 (4.81 - 6.63)** | -0.39 (-1.64 - 0.86) | **0.29 (0.24 - 0.35)** | 0.92 (0.69 - 1.23) |
| Sex | | | | |
| Male (Reference: Female) | | -0.77 (-1.72 - 0.19) | | 0.86 (0.72 - 1.03) |
| Age group | | | | |
| 45-64 (Reference 18-44 years) | | -0.99 (-2.13 - 0.15) | | 1.14 (0.92 - 1.43) |
| 65+ | | **-4.22 (-5.42 - -3.03)** | | 1.04 (0.83 - 1.31) |
| Comorbidity | | | | |
| DM (Reference: no DM) | | 6.33 (1.36 - 11.31) | | 0.65 (0.22 - 1.88) |
| Years since diagnosis | | 0.12 (-0.02 - 0.25) | | 1.00 (0.97 - 1.02) |
| Time in the programme | | **-1.67 (-1.99 - -1.35)** | | **1.33 (1.25 - 1.42)** |
| Co-location of NCD/HIV services | | | | |
| Co-located (Reference: located separately) | | **-5.28 (-7.32 - -3.24)** | | 1.16 (0.77 - 1.73) |
| Covid index | | **5.67 (4.61 - 6.73)** | | **0.40 (0.31 - 0.51)** |
| Proportion of new patients per month | | **-2.43 (-4.48 - -0.39)** | | 1.58 (0.97 - 2.59) |
| Restricted to sites that implemented all seven activities and excluding first two visits ($n_s$=10; $n_v$=9,927) | Beta (95% CI) | aBeta (95% CI)[a] | Beta (95% CI) | Beta (95% CI)[a] |
| PIC4C implementation index | 3.95 (2.88-5.01) | -0.59 (-2.03 - 0.85) | **0.43 (0.34 - 0.55)** | 0.93 (0.66 - 1.32) |
| Sex | | | | |
| Male (Reference: Female) | | -1.08 (-2.29 - 0.13) | | 0.89 (0.71 - 1.13) |
| Age group | | | | |
| 45-64 (Reference 18-44 years) | | -0.24 (-1.65 - 1.17) | | 1.00 (0.75 - 1.33) |
| 65+ | | **-2.66 (-4.15 - -1.18)** | | 0.92 (0.69 - 1.24) |
| Comorbidity | | | | |
| DM (Reference: no DM) | | 7.88 (-15.64 - 31.39) | | 1.00 (0.90 - 1.11) |
| Years since diagnosis | | **0.23 (0.08 - 0.39)** | | 0.98 (0.95 - 1.01) |
| Time in the programme | | **-0.66 (-1.07 - -0.25)** | | **1.14 (1.05 - 1.24)** |
| Co-location of NCD/HIV services | | | | |
| Co-located (Reference: located separately) | | -2.18 (-4.95 - 0.59) | | 0.88 (0.51 - 1.52) |
| Covid index | | **5.33 (4.16 - 6.50)** | | **0.47 (0.35 - 0.64)** |
| Proportion of new patients per month | | **-2.64 (-4.94 - -0.34)** | | 1.68 (0.95 - 2.97) |

aBeta Adjusted beta coefficient; aOR Adjusted Odds Ratio; CI Confidence interval; DBP Diastolic blood pressure; DM Diabetes mellitus; NCD: Non-communicable diseases; $n_s$ Number of sites; $n_v$ Number of visits; SBP Systolic blood pressure. P<0.05 highlighted in bold.

[a] Mixed effects models using random effects for patients and fixed effects for sites.

**Table S4b:** Impact of PIC4C implementation on random plasma glucose and diabetes control.

| | RPG (mmol/L) | | DM control | |
|---|---|---|---|---|
| All visits and sites (RPG: $n_v$=15,778; DM control: $n_v$=24,005) | Beta (95% CI) | aBeta (95% CI)[a] | OR (95% CI) | aOR (95% CI)[a] |
| PIC4C implementation index | **1.96 (1.49 - 2.43)** | 0.46 (-0.13 - 1.05) | **0.39 (0.32 - 0.46)** | **0.59 (0.47 - 0.73)** |
| Sex | | | | |
| Male (Reference: Female) | | 0.30 (0.04 - 0.56) | | 0.97 (0.87 - 1.09) |
| Age group | | | | |
| 45-64 (Reference 18-44 years) | | **-0.84 (-1.17 - -0.51)** | | **1.40 (1.21 - 1.60)** |
| 65+ | | **-1.40 (-1.76 - -1.05)** | | **1.84 (1.58 - 2.13)** |
| Comorbidity | | | | |
| HTN (Reference: no HTN) | | **-1.16 (-1.45 - -0.87)** | | **1.49 (1.32 - 1.69)** |
| Years since diagnosis | | **0.12 (0.09 - 0.16)** | | **0.93 (0.91 - 0.94)** |
| Time in the programme | | -0.07 (-0.17 - 0.04) | | **1.12 (1.07 - 1.17)** |
| Co-location of NCD/HIV services | | | | |
| Co-located (Reference: located separately) | | -0.32 (-1.59 - 0.95) | | 0.90 (0.60 - 1.33) |
| Covid index | | **1.35 (0.85 - 1.85)** | | **0.81 (0.66 - 0.98)** |
| Proportion of new patients per month | | 0.00 (-1.05 - 1.05) | | **1.46 (1.01 - 2.09)** |
| Excluding first two visits (RPG: $n_v$=9,202; DM control: $n_v$=15,402) | Beta (95% CI) | aBeta (95% CI)[a] | OR (95% CI) | aOR (95% CI)[a] |
| PIC4C implementation index | **1.46 (0.82 - 2.11)** | 0.43 (-0.31 - 1.17) | **0.45 (0.36 - 0.58)** | **0.62 (0.47 - 0.82)** |
| Sex | | | | |
| Male (Reference: Female) | | -0.03 (-0.38 - 0.32) | | 1.07 (0.92 - 1.25) |
| Age group | | | | |
| 45-64 (Reference 18-44 years) | | -0.32 (-0.77 - 0.14) | | **1.34 (1.11 - 1.63)** |
| 65+ | | **-0.71 (-1.19 - -0.23)** | | **1.70 (1.38 - 2.09)** |
| Comorbidity | | | | |
| HTN (Reference: no HTN) | | -0.43 (-0.87 - 0.02) | | 1.10 (0.91 - 1.33) |
| Years since diagnosis | | 0.11 (0.06 - 0.15) | | **0.93 (0.91 - 0.94)** |
| Time in the programme | | **-0.34 (-0.49 - -0.19)** | | **1.28 (1.20 - 1.37)** |
| Co-location of NCD/HIV services | | | | |
| Co-located (Reference: located separately) | | -0.47 (-1.89 - 0.96) | | 0.88 (0.56 - 1.38) |
| Covid index | | **0.69 (0.12 - 1.27)** | | 1.05 (0.84 - 1.33) |
| Proportion of new patients per month | | 0.09 (-1.16 - 1.34) | | 1.39 (0.90 - 2.15) |
| Restricted to sites that implemented all seven activities ($n_s$=10; RPG: $n_v$=2,279; DM control: $n_v$=4,358) | Beta (95% CI) | aBeta (95% CI)[a] | Beta (95% CI) | Beta (95% CI)[a] |
| PIC4C implementation index | **1.84 (0.88 - 2.81)** | -0.03 (-1.24 - 1.19) | **0.23 (0.16 - 0.35)** | 0.64 (0.38 - 1.09) |
| Sex | | | | |
| Male (Reference: Female) | | 0.23 (-0.45 - 0.90) | | 1.07 (0.76 - 1.52) |
| Age group | | | | |
| 45-64 (Reference 18-44 years) | | -0.00 (-0.85 - 0.85) | | 1.24 (0.81 - 1.89) |
| 65+ | | -0.75 (-1.65 - 0.15) | | **1.84 (1.17 - 2.90)** |
| Comorbidity | | | | |
| HTN (Reference: no HTN) | | -0.61 (-1.35 - 0.13) | | 1.13 (0.78 - 1.63) |
| Years since diagnosis | | **0.20 (0.11 - 0.29)** | | **0.84 (0.80 - 0.89)** |
| Time in the programme | | **-0.26 (-0.52 - -0.01)** | | **1.49 (1.30 - 1.70)** |
| Co-location of NCD/HIV services | | | | |
| Co-located (Reference: located separately) | | -0.91 (-2.66 - 0.83) | | 0.41 (0.17 - 1.01) |
| Covid index | | **1.94 (0.88 - 3.00)** | | **0.59 (0.37 - 0.94)** |
| Proportion of new patients per month | | -0.78 (-3.02 - 1.46) | | 0.92 (0.34 - 2.49) |
| Restricted to sites that implemented all seven activities and excluding first two visits ($n_s$=10; RPG: $n_v$=1,455; DM control: $n_v$=3,064) | Beta (95% CI) | aBeta (95% CI)[a] | Beta (95% CI) | aBeta (95% CI)[a] |
| PIC4C implementation index | **2.15 (0.76 - 3.54)** | 0.70 (-0.90 - 2.31) | **0.25 (0.15 - 0.42)** | **0.49 (0.26 - 0.95)** |
| Sex | | | | |
| Male (Reference: Female) | | 0.14 (-0.77 - 1.05) | | 1.19 (0.74 - 1.91) |
| Age group | | | | |
| 45-64 (Reference 18-44 years) | | 0.21 (-0.89 - 1.30) | | 1.34 (0.77 - 2.33) |
| 65+ | | -0.63 (-1.80 - 0.54) | | **2.10 (1.16 - 3.80)** |
| Comorbidity | | | | |
| HTN (Reference: no HTN) | | -0.78 (-1.91 - 0.35) | | 1.06 (0.62 - 1.80) |
| Years since diagnosis | | **0.21 (0.10 - 0.32)** | | **0.83 (0.78 - 0.88)** |
| Time in the programme | | **-0.50 (-0.84 - -0.16)** | | **1.59 (1.33 - 1.92)** |
| Co-location of NCD/HIV services | | | | |
| Co-located (Reference: located separately) | | -0.95 (-4.10 - 2.20) | | 0.48 (0.11 - 2.06) |
| Covid index | | 1.08 (-0.16 - 2.32) | | 0.82 (0.47 - 1.41) |
| Proportion of new patients per month | | -0.09 (-2.78 - 2.61) | | 1.24 (0.38 - 4.05) |

aBeta Adjusted beta coefficient; aOR Adjusted Odds Ratio; BP Blood pressure; CI Confidence interval; DM Diabetes mellitus; NCD: Non-communicable diseases; $n_s$ Number of sites; $n_v$ Number of visits; RPG Random plasma glucose. P<0.05 highlighted in bold.

[a] Mixed effects models using random effects for patients and fixed effects for sites.

**Table S5:** Impact of individual PIC4C implementation components on hypertension, diabetes and viral suppression.

| | BP control | | DBP | | DM control | | RPG | | Viral suppression | |
|---|---|---|---|---|---|---|---|---|---|---|
| | OR (95% CI) | aOR (95% CI)[a] | Beta (95% CI) | Beta (95% CI)[a] | OR (95% CI) | aOR (95% CI)[b] | Beta (95% CI) | Beta (95% CI)[b] | OR (95% CI) | aOR (95% CI)[c] |
| **All visits and sites** | **$n_v$=66,641** | | **$n_v$=66,641** | | **$n_v$=24,005** | | **$n_v$=15,778** | | **$n_v$=84,855** | |
| Training | 0.77 (0.73-0.81) | 1.07 (1.01-1.14) | 0.34 (0.08-0.59) | -1.08 (-1.35--0.82) | 0.70 (0.64-0.77) | 0.86 (0.78-0.96) | 0.75 (0.51-0.99) | 0.13 (-0.14-0.39) | 1.14 (1.05-1.23) | 1.02 (0.93-1.11) |
| RFPS | 0.43 (0.40-0.46) | 0.66 (0.61-0.72) | 5.16 (4.86-5.46) | 3.50 (3.15-3.85) | 0.53 (0.46-0.61) | 0.67 (0.57-0.77) | 1.42 (0.84-2.00) | 0.72 (0.13-1.32) | 1.01 (0.91-1.13) | 0.85 (0.75-0.96) |
| Mentorship | 0.64 (0.61-0.68) | 0.96 (0.90-1.02) | 1.48 (1.23-1.72) | -0.25 (-0.51-0.02) | 0.67 (0.61-0.73) | 0.84 (0.76-0.93) | 0.71 (0.48-0.94) | 0.13 (-0.13-0.38) | 1.28 (1.19-1.39) | 1.15 (1.05-1.26) |
| Data strengthening | 0.58 (0.55-0.61) | 0.93 (0.87-1.00) | 2.45 (2.20-2.71) | 0.12 (-0.18-0.43) | 0.63 (0.58-0.69) | 0.79 (0.71-0.88) | 1.05 (0.81-1.30) | 0.38 (0.09-0.67) | 1.20 (1.11-1.30) | 1.03 (0.93-1.14) |
| Patient support group | 0.47 (0.44-0.50) | 0.72 (0.66-0.78) | 3.60 (3.34-3.87) | 1.62 (1.26-1.97) | 0.59 (0.54-0.66) | 0.71 (0.62-0.82) | 1.20 (0.93-1.46) | 0.39 (0.01-0.76) | 1.45 (1.33-1.57) | 1.29 (1.13-1.48) |
| Equipment | 0.94 (0.59-1.50) | 1.29 (0.80-2.08) | -0.32 (-2.41-1.77) | -1.87 (-3.93-0.19) | 1.73 (0.84-3.56) | 2.24 (1.09-4.62) | -0.99 (-2.49-0.52) | -1.65 (-3.15--0.15) | -- | -- |
| Group cares | 1.05 (0.94-1.18) | 1.08 (0.97-1.21) | 0.67 (0.17-1.17) | 0.68 (0.18-1.18) | 1.30 (1.08-1.57) | 1.33 (1.10-1.60) | -0.64 (-1.10--0.18) | -0.64 (-1.10--0.19) | 1.18 (0.98-1.42) | 1.19 (0.99-1.43) |
| **Excluding first two visits** | **($n_v$=44,901)** | | **($n_v$=44,901)** | | **($n_v$=15,402)** | | **($n_v$=9,202)** | | | |
| Training | 0.95 (0.89-1.01) | 1.11 (1.03-1.19) | -0.26 (-0.54-0.02) | -0.89 (-1.19--0.59) | 0.79 (0.70-0.90) | 0.92 (0.81-1.04) | 0.42 (0.11-0.73) | 0.04 (-0.29-0.37) | | |
| RFPS | 0.56 (0.52-0.61) | 0.69 (0.63-0.76) | 3.79 (3.46-4.11) | 2.95 (2.57-3.33) | 0.62 (0.53-0.73) | 0.71 (0.60-0.85) | 0.94 (0.27-1.61) | 0.54 (-0.14-1.23) | | |
| Mentorship | 0.82 (0.77-0.87) | 0.99 (0.92-1.06) | 0.77 (0.49-1.05) | -0.01 (-0.30-0.29) | 0.69 (0.61-0.78) | 0.81 (0.71-0.92) | 0.60 (0.30-0.90) | 0.22 (-0.10-0.54) | | |
| Data strengthening | 0.76 (0.71-0.82) | 1.01 (0.93-1.10) | 1.33 (1.05-1.62) | -0.14 (-0.48-0.20) | 0.72 (0.63-0.81) | 0.84 (0.73-0.96) | 0.69 (0.38-1.01) | 0.27 (-0.08-0.62) | | |
| Patient support group | 0.63 (0.59-0.67) | 0.79 (0.72-0.87) | 2.53 (2.24-2.83) | 1.36 (0.97-1.74) | 0.69 (0.60-0.78) | 0.75 (0.64-0.88) | 0.66 (0.32-1.00) | 0.11 (-0.32-0.54) | | |
| Equipment | 1.16 (0.60-2.26) | 1.39 (0.72-2.71) | -0.70 (-3.59-2.19) | -1.74 (-4.61-1.13) | 1.42 (0.52-3.92) | 1.71 (0.63-4.67) | -0.44 (-2.39-1.51) | -0.90 (-2.84-1.05) | | |
| Group cares | 0.94 (0.80-1.10) | 0.95 (0.81-1.11) | 1.07 (0.39-1.75) | 1.01 (0.33-1.68) | 1.55 (1.18-2.04) | 1.54 (1.17-2.02) | -0.36 (-1.02-0.30) | -0.33 (-0.99-0.33) | | |
| **Restricted to sites that implemented all seven activities (n=10)** | **($n_v$=13,625)** | | **($n_v$=13,625)** | | **($n_v$=4,358)** | | **($n_v$=2,279)** | | | |
| Training | 0.63 (0.57-0.71) | 1.12 (0.96-1.29) | 1.39 (0.85-1.92) | -1.13 (-1.77--0.50) | 0.62 (0.49-0.78) | 0.99 (0.76-1.29) | 0.47 (-0.08-1.01) | -0.38 (-0.99-0.23) | | |
| RFPS | 0.44 (0.38-0.49) | 0.78 (0.66-0.93) | 3.98 (3.41-4.55) | 0.96 (0.23-1.70) | 0.33 (0.25-0.44) | 0.55 (0.39-0.77) | 2.36 (1.49-3.24) | 1.43 (0.49-2.37) | | |
| Mentorship | 0.48 (0.43-0.54) | 0.92 (0.78-1.08) | 3.29 (2.76-3.83) | 0.03 (-0.67-0.72) | 0.51 (0.40-0.64) | 0.91 (0.68-1.23) | 1.05 (0.50-1.59) | 0.13 (-0.53-0.79) | | |
| Data strengthening | 0.51 (0.46-0.57) | 0.96 (0.82-1.12) | 2.49 (1.96-3.02) | -0.69 (-1.38--0.01) | 0.54 (0.43-0.69) | 0.91 (0.70-1.20) | 0.84 (0.30-1.37) | -0.00 (-0.62-0.62) | | |
| Patient support group | 0.42 (0.38-0.48) | 0.75 (0.61-0.91) | 3.83 (3.30-4.36) | 0.11 (-0.71-0.93) | 0.41 (0.32-0.52) | 0.69 (0.49-0.97) | 1.29 (0.74-1.84) | 0.22 (-0.56-1.00) | | |
| Equipment* | -- | -- | -- | -- | -- | -- | -- | -- | | |

| | | | | | | | | |
|---|---|---|---|---|---|---|---|---|
| Group cares | 1.45 (1.18-1.79) | 1.53 (1.22-1.91) | -0.05 (-0.92-0.81) | 0.45 (-0.48-1.38) | 0.97 (0.67-1.40) | 0.98 (0.68-1.43) | -0.64 (-1.31-0.03) | -0.64 (-1.32-0.04) |
| **Restricted to sites that implemented all seven activities (n=10) and excluding first two visits** | **($n_v$=9,927)** | | **($n_v$=9,927)** | | **($n_v$=3,064)** | | **($n_v$=1,455)** | |
| Training | 0.87 (0.75-1.00) | 1.21 (1.02-1.44) | 0.19 (-0.41-0.80) | -0.99 (-1.71--0.27) | 0.71 (0.53-0.95) | 0.94 (0.68-1.29) | 0.27 (-0.45-1.00) | -0.29 (-1.07-0.48) |
| RFPS | 0.53 (0.46-0.62) | 0.74 (0.60-0.90) | 3.24 (2.60-3.87) | 1.05 (0.24-1.86) | 0.36 (0.26-0.51) | 0.49 (0.33-0.73) | 2.44 (1.33-3.55) | 1.70 (0.55-2.85) |
| Mentorship | 0.65 (0.56-0.75) | 1.03 (0.86-1.25) | 2.15 (1.53-2.76) | -0.27 (-1.04-0.50) | 0.55 (0.41-0.75) | 0.81 (0.57-1.15) | 1.15 (0.43-1.88) | 0.56 (-0.26-1.37) |
| Data strengthening | 0.66 (0.58-0.76) | 1.02 (0.85-1.23) | 1.45 (0.85-2.05) | -0.72 (-1.48-0.04) | 0.59 (0.44-0.79) | 0.81 (0.59-1.11) | 0.88 (0.16-1.59) | 0.34 (-0.43-1.11) |
| Patient support group | 0.53 (0.46-0.62) | 0.76 (0.60-0.95) | 3.05 (2.44-3.66) | 0.15 (-0.76-1.07) | 0.46 (0.34-0.62) | 0.63 (0.43-0.95) | 1.02 (0.28-1.75) | 0.15 (-0.78-1.07) |
| Equipment* | -- | -- | -- | -- | -- | -- | -- | -- |
| Group cares | 1.30 (0.81-2.07) | 1.27 (0.79-2.05) | -1.14 (-3.03-0.74) | -0.63 (-2.55-1.29) | 1.39 (0.68-2.84) | 1.26 (0.62-2.58) | -0.89 (-2.20-0.42) | -0.82 (-2.15-0.50) |

BP Blood pressure; CI Confidence interval; DBP Diastolic blood pressure; DM Diabetes mellitus; FPG Fasting plasma glucose; $n_v$ Number of visits; RPG Random plasma glucose; SBP Systolic blood pressure.

*All facilities had equipment as of January 2017.

[a] Mixed effects models using random effects for patients and fixed effects for sites adjusted for sex, age, diabetes, time in the programme, time since hypertension diagnosis, services co-location, impact of covid policies that primarily restrict people's behaviour, proportion of new patients with hypertension per month and seasonality.

[b] Mixed effects models using random effects for patients and fixed effects for sites adjusted for sex, age, hypertension, time in the programme, time since diabetes diagnosis, services co-location, impact of covid policies that primarily restrict people's behaviour, and proportion of new patients with diabetes per month.

[c] Mixed effects models using random effects for patients and fixed effects for sites adjusted for sex, age, comorbidity (diabetes, hypertension or both), time in the programme, services co-location, impact of covid policies that primarily restrict people's behaviour and proportion of new patients with hypertension or diabetes per month.

**Table S6:** Impact of PIC4C implementation on viral load.

| | Viral load (copies/mL) | | Viral suppression | |
|---|---|---|---|---|
| **All visits and sites ($n_s$=14; $n_v$=84,855)** | **Beta (95% CI)** | **aBeta (95% CI)[a]** | **OR** | **aOR[a]** |
| **PIC4C implementation index** | 2060.09 (923.09-3197.08) | -1573.35 (-3163.04-16.33) | 1.58 (1.36-1.83) | 1.20 (0.98-1.47) |
| **Sex** | | | | |
| Male (Reference: Female) | | 1825.20 (1153.26-2497.15) | | 0.66 (0.61-0.71) |
| **Age group** | | | | |
| 45-64 (Reference 18-44 years) | | -1755.51 (-2371.32--1139.69) | | 1.29 (1.19-1.39) |
| 65+ | | -1043.73 (-2609.18-521.72) | | 1.35 (1.11-1.66) |
| **Comorbidity** | | | | |
| HTN/DM (Reference: no HTN/DM) | | -1108.90 (-3804.77-1586.96) | | 1.32 (0.93-1.88) |
| DM | | -1693.34 (-4624.22-1237.53) | | 1.24 (0.85-1.80) |
| HTN | | -2536.67 (-16499.04-11425.71) | | 1.26 (0.21-7.53) |
| **Time in the programme** | | -1151.08 (-1492.10--810.06) | | 0.99 (0.96-1.03) |
| **Covid index** | | 1598.43 (485.80-2711.06) | | 1.34 (1.14-1.57) |
| **Proportion of new patients** | | **-2334.07 (-4081.93 - -586.21)** | | 2.84 (2.20-3.67) |

aBeta Adjusted beta coefficient; aOR Adjusted Odds Ratio; BP Blood pressure; CI Confidence interval; DBP Diastolic blood pressure; DM Diabetes mellitus; FPG Fasting plasma glucose; $n_s$ Number of sites; $n_v$ Number of visits; RPG Random plasma glucose; SBP Systolic blood pressure.

[a] Mixed effects models using random effects for patients and fixed effects for sites adjusted for sex, age, comorbidity (diabetes, hypertension or both), time in the programme and impact of covid policies that primarily restrict people's behaviour.

**Table S7:** Impact of PIC4C implementation on systolic blood pressure and fasting plasma glucose).

| | SBP (mm HG) | | FPG (mmol/L) | |
|---|---|---|---|---|
| **Excluding first two visits ($n_v$=44,901)** | **Beta (95% CI)** | **Beta (95% CI)[a]** | **Beta (95% CI)** | **Beta (95% CI)[a]** |
| **PIC4C implementation index** | **5.02 (4.08 - 5.95)** | **1.39 (0.28 - 2.49)** | **1.42 (0.93 - 1.91)** | **0.72 (0.12 - 1.32)** |
| **Sex** | | | | |
| Male (Reference: Female) | | **1.48 (0.66 - 2.29)** | | -0.28 (-0.66 - 0.09) |
| **Age group** | | | | |
| 45-64 (Reference 18-44 years) | | 0.68 (-0.30 - 1.65) | | **-0.83 (-1.27 - -0.38)** |
| 65+ | | **3.88 (2.85 - 4.92)** | | **-1.22 (-1.71 - -0.74)** |
| **Comorbidity** | | | | |
| DM (Reference: no DM) | | -6.94 (-16.41 - 2.54) | | -0.37 (-0.79 - 0.06) |
| HTN (Reference: no HTN) | | | | |
| **Years since diagnosis** | | **0.16 (0.08 - 0.25)** | | **0.10 (0.06 - 0.14)** |
| **Time in the programme** | | **-0.99 (-1.30 - -0.68)** | | **-0.47 (-0.63 - -0.31)** |
| **Co-location of NCD/HIV services** | | | | |
| Co-located (Reference: located separately) | | -1.41 (-3.01 - 0.18) | | -0.19 (-1.02 - 0.64) |
| **Covid index** | | **4.14 (3.26 - 5.02)** | | 0.21 (-0.32 - 0.74) |
| **Proportion of new patients per month** | | 1.11 (-0.32 - 2.53) | | -0.65 (-1.54 - 0.24) |
| **Restricted to sites that implemented all seven activities ($n_s$=10; $n_v$=13,625)** | **Beta (95% CI)** | **aBeta (95% CI)[a]** | **Beta (95% CI)** | **aBeta (95% CI)[a]** |
| **PIC4C implementation index** | **8.97 (7.46 - 10.48)** | 0.95 (-1.17 - 3.07) | **2.12 (1.40 - 2.83)** | 0.42 (-0.59 - 1.44) |
| **Sex** | | | | |
| Male (Reference: Female) | | 0.94 (-0.60 - 2.49) | | -0.32 (-0.96 - 0.33) |
| **Age group** | | | | |
| 45-64 (Reference 18-44 years) | | -0.88 (-2.74 - 0.99) | | **-0.95 (-1.74 - -0.16)** |
| 65+ | | **2.51 (0.56 - 4.46)** | | **-1.88 (-2.72 - -1.04)** |
| **Comorbidity** | | | | |
| DM (Reference: no DM) | | 1.56 (-6.69 - 9.81) | | -0.35 (-1.03 - 0.32) |
| HTN (Reference: no HTN) | | | | |
| **Years since diagnosis** | | 0.06 (-0.15 - 0.28) | | **0.17 (0.09 - 0.25)** |
| **Time in the programme** | | **-2.71 (-3.23 - -2.19)** | | **-0.57 (-0.82 - -0.31)** |
| **Co-location of NCD/HIV services** | | | | |
| Co-located (Reference: located separately) | | -0.15 (-3.46 - 3.16) | | 2.55 (-0.50 - 5.59) |
| **Covid index** | | **5.84 (4.04 - 7.65)** | | 0.90 (-0.05 - 1.85) |
| **Proportion of new patients per month** | | -1.10 (-4.59 - 2.39) | | **-2.17 (-4.11 - -0.23)** |

aBeta Adjusted beta coefficient; CI Confidence interval; DBP Diastolic blood pressure; DM Diabetes mellitus; NCD: Non-communicable diseases; $n_s$ Number of sites; $n_v$ Number of visits; SBP Systolic blood pressure. P<0.05 highlighted in bold.

[a] Mixed effects models using random effects for patients and fixed effects for sites.

**Table S8:** Impact of individual PIC4C implementation on systolic blood pressure and fasting plasma glucose.

| | **SBP** | | **FPG** | |
|---|---|---|---|---|
| **Excluding first two visits** | **($n_v$=44,901)** | | **($n_v$=6,217)** | |
| Training | **0.49 (0.01-0.97)** | -0.46 (-0.98-0.05) | **0.39 (0.11-0.66)** | 0.11 (-0.18-0.40) |
| RFPS | **3.79 (3.24-4.35)** | **2.11 (1.46-2.75)** | **0.87 (0.58-1.16)** | **0.56 (0.20-0.92)** |
| Mentorship | **1.71 (1.23-2.19)** | **0.52 (0.01-1.03)** | **0.66 (0.38-0.94)** | 0.26 (-0.05-0.57) |
| Data strengthening | **1.97 (1.49-2.46)** | -0.24 (-0.82-0.34) | **0.59 (0.32-0.86)** | 0.20 (-0.10-0.50) |
| Patient support group | **3.42 (2.92-3.93)** | **1.16 (0.50-1.83)** | **0.88 (0.60-1.17)** | **0.62 (0.25-0.98)** |
| Equipment | 0.45 (-4.48-5.38) | -0.74 (-5.65-4.17) | 5.35 (-1.19-11.89) | 5.69 (-0.74-12.13) |
| Group cares | 0.89 (-0.27-2.05) | 1.04 (-0.11-2.20) | **-0.70 (-1.38 - -0.02)** | -0.61 (-1.29-0.07) |
| **Restricted to sites that implemented all seven activities (n=10)** | **($n_v$=13,625)** | | **($n_v$=2,082)** | |
| Training | **3.00 (2.11-3.89)** | **-1.12 (-2.19--0.04)** | **0.81 (0.38-1.24)** | -0.06 (-0.56-0.43) |
| RFPS | **5.89 (4.93-6.85)** | **1.88 (0.64-3.13)** | **1.38 (0.93-1.83)** | 0.53 (-0.14-1.19) |
| Mentorship | **5.10 (4.21-6.00)** | 0.64 (-0.54-1.82) | **1.12 (0.66-1.58)** | 0.07 (-0.52-0.66) |
| Data strengthening | **4.81 (3.93-5.69)** | 0.78 (-0.39-1.95) | **1.14 (0.71-1.57)** | 0.25 (-0.28-0.77) |
| Patient support group | **6.15 (5.26-7.04)** | **2.51 (1.10-3.91)** | **1.39 (0.95-1.84)** | 0.55 (-0.11-1.22) |
| Equipment* | -- | -- | -- | -- |
| Group cares | **-2.27 (-3.74--0.80)** | **-2.67 (-4.25--1.09)** | -1.18 (-2.60-0.25) | -0.81 (-2.21-0.59) |

BP Blood pressure; CI Confidence interval; DBP Diastolic blood pressure; DM Diabetes mellitus; FPG Fasting plasma glucose; $n_v$ Number of visits; RPG Random plasma glucose; SBP Systolic blood pressure.

P<0.05 highlighted in bold.

*All facilities had equipment as of January 2017.

[a] Mixed effects models using random effects for patients and fixed effects for sites adjusted for sex, age, diabetes, time in the programme, time since hypertension diagnosis, services co-location, impact of covid policies that primarily restrict people's behaviour, proportion of new patients with hypertension per month and seasonality.

[b] Mixed effects models using random effects for patients and fixed effects for sites adjusted for sex, age, hypertension, time in the programme, time since diabetes diagnosis, services co-location, impact of covid policies that primarily restrict people's behaviour, and proportion of new patients with diabetes per month.

[c] Mixed effects models using random effects for patients and fixed effects for sites adjusted for sex, age, comorbidity (diabetes, hypertension or both), time in the programme, services co-location, impact of covid policies that primarily restrict people's behaviour and proportion of new patients with hypertension or diabetes per month

**Table S9:** Impact of PIC4C implementation index on hypertension, diabetes and viral suppression using unequal weights.

| | **Primary outcomes** | | | | **Secondary outcomes** | | | | | | | | | | | |
|---|---|---|---|---|---|---|---|---|---|---|---|---|---|---|---|---|
| | **SBP** | | **FPG** | | **BP control** | | **DBP** | | **DM control** | | **RPG** | | **Viral suppression** | | **Viral load (copies/mL)** | |
| | **Beta (95% CI)** | **Beta (95% CI)[a]** | **Beta (95% CI)** | **Beta (95% CI)[b]** | **OR (95% CI)** | **aOR (95% CI)[a]** | **Beta (95% CI)** | **Beta (95% CI)[a]** | **OR (95% CI)** | **aOR (95% CI)[b]** | **Beta (95% CI)** | **Beta (95% CI)[b]** | **OR (95% CI)** | **aOR (95% CI)[c]** | **Beta (95% CI)** | **Beta (95% CI)[c]** |
| **All visits and sites** | **$n_v$=66,641** | | | | **$n_v$=8,268** | | | | **$n_v$=24,005** | | **$n_v$=15,778** | | **$n_v$=84,855** | | **$n_v$=84,855** | |
| Implementation index (Unequal weights)* | 10.60 (9.78-11.43) | 2.62 (1.59-3.66) | 1.52 (1.11-1.94) | 0.65 (0.10-1.20) | 0.28 (0.25-0.31) | 0.67 (0.58-0.77) | 6.58 (6.09-7.06) | 2.54 (1.93-3.14) | 0.36 (0.30-0.44) | 0.55 (0.44-0.70) | 2.19 (1.67-2.72) | 0.49 (-0.18-1.16) | 1.65 (1.41-1.92) | 1.22 (0.97-1.52) | 2318 (1133-3503) | -1443 (-3173-286) |
| **Excluding first two visits** | **($n_v$=44,901)** | | | | **($n_v$=6,217)** | | | | **($n_v$=15,402)** | | **($n_v$=9,202)** | | | | | |
| Implementation index (Unequal weights)* | 5.82 (4.87-6.77) | 2.19 (1.03-3.35) | 1.45 (0.96-1.93) | 0.80 (0.19-1.41) | 0.46 (0.40-0.52) | 0.73 (0.62-0.86) | 4.48 (3.92-5.03) | 2.39 (1.71-3.06) | 0.45 (0.35-0.57) | 0.60 (0.44-0.80) | 1.61 (0.90-2.32) | 0.45 (-0.38-1.29) | | | | |
| **Restricted to sites that implemented all seven activities (n=10)** | **($n_v$=13,625)** | | | | **($n_v$=2,082)** | | | | **($n_v$=4,358)** | | **($n_v$=2,279)** | | | | | |
| Implementation index (Unequal weights)* | 9.34 (7.82-10.86) | 1.36 (-0.81-3.53) | 2.16 (1.45-2.88) | 0.54 (-0.51-1.58) | 0.28 (0.23-0.34) | 0.88 (0.66-1.19) | 6.13 (5.21-7.04) | 0.12 (-1.16-1.40) | 0.21 (0.14-0.31) | 0.55 (0.32-0.96) | 2.02 (1.01-3.04) | 0.08 (-1.21-1.36) | | | | |
| **Restricted to sites that implemented all seven activities (n=10) and excluding first two visits** | **($n_v$=9,927)** | | | | **($n_v$=1,610)** | | | | **($n_v$=3,064)** | | **($n_v$=1,455)** | | | | | |
| Implementation index (Unequal weights)* | 6.43 (4.63-8.23) | 2.80 (0.29-5.31) | 2.03 (1.13-2.92) | 0.75 (-0.44-1.94) | 0.40 (0.31-0.51) | 0.81 (0.57-1.16) | 4.44 (3.37-5.51) | -0.09 (-1.57-1.40) | 0.22 (0.13-0.38) | 0.42 (0.21-0.83) | 2.45 (0.97-3.93) | 0.86 (-0.87-2.59) | | | | |

BP Blood pressure; CI Confidence interval; DBP Diastolic blood pressure; DM Diabetes mellitus; FPG Fasting plasma glucose; $n_v$ Number of visits; RPG Random plasma glucose; SBP Systolic blood pressure.

*20% weight on RFPS, Group cares and patient support group. 10% weight on training, equipment, mentorship and data strengthening.

[a] Mixed effects models using random effects for patients and fixed effects for sites adjusted for sex, age, diabetes, time in the programme, time since hypertension diagnosis, services co-location, impact of covid policies that primarily restrict people's behaviour, proportion of new patients with hypertension per month and seasonality.

[b] Mixed effects models using random effects for patients and fixed effects for sites adjusted for sex, age, hypertension, time in the programme, time since diabetes diagnosis, services co-location, impact of covid policies that primarily restrict people's behaviour, and proportion of new patients with diabetes per month.

[c] Mixed effects models using random effects for patients and fixed effects for sites adjusted for sex, age, comorbidity (diabetes, hypertension or both), time in the programme, services co-location, impact of covid policies that primarily restrict people's behaviour and proportion of new patients with hypertension or diabetes per month.
